# Indigo Carmine Binding to Cu(II) in Aqueous Solution and Solid State: Full Structural Characterization Using NMR, FTIR and UV/Vis Spectroscopies and DFT Calculations

**DOI:** 10.3390/molecules29133223

**Published:** 2024-07-07

**Authors:** Sofia Braz, Licínia L. G. Justino, M. Luísa Ramos, Rui Fausto

**Affiliations:** 1 CQC-IMS, Department of Chemistry, University of Coimbra, Rua Larga, 3004-535 Coimbra, Portugal; 2 CERES, Department of Chemical Engineering, University of Coimbra, Pólo II, Rua Sílvio Lima, 3030-790 Coimbra, Portugal; 3 Istanbul Kultur University, Faculty of Sciences and Letters, Department of Physics, 34158 Bakirkoy, Istanbul, Turkey

**Keywords:** indigo carmine, Cu^2+^, metal complexes, DFT, NMR, FTIR, UV/Vis

## Abstract

The food industry uses indigo carmine (IC) extensively as a blue colorant to make processed food for young children and the general population more attractive. Given that IC can act as a ligand, this raises concerns about its interactions with essential metal ions in the human body. In view of this interest, in the present investigation, the copper(II)/indigo carmine system was thoroughly investigated in aqueous solution and in the solid state, and the detailed structural characterization of the complexes formed between copper(II) and the ligand was performed using spectroscopic methods, complemented with DFT and TD-DFT calculations. NMR and UV/Vis absorption spectroscopy studies of the ligand in the presence of copper(II) show changes that clearly reveal strong complexation. The results point to the formation of complexes of 1:1, 1:2 and 2:1 Cu(II)/IC stoichiometry in aqueous solution, favored in the pH range 6–10 and stable over time. DFT calculations indicate that the coordination of the ligand to the metal occurs through the adjacent carbonyl and amine groups and that the 1:1 and the 2:1 complexes have distorted tetrahedral metal centers, while the 1:2 structure is five-coordinate with a square pyramidal geometry. FTIR results, together with EDS data and DFT calculations, established that the complex obtained in the solid state likely consists of a polymeric arrangement involving repetition of the 1:2 complex unit. These results are relevant in the context of the study of the toxicity of IC and provide crucial data for future studies of its physiological effects. Although the general population does not normally exceed the maximum recommended daily intake, young children are highly exposed to products containing IC and can easily exceed the recommended dose. It is, therefore, extremely important to understand the interactions between the dye and the various metal ions present in the human body, copper(II) being one of the most relevant due to its essential nature and, as shown in this article, the high stability of the complexes it forms with IC at physiological pH.

## 1. Introduction

**Scheme 1:**
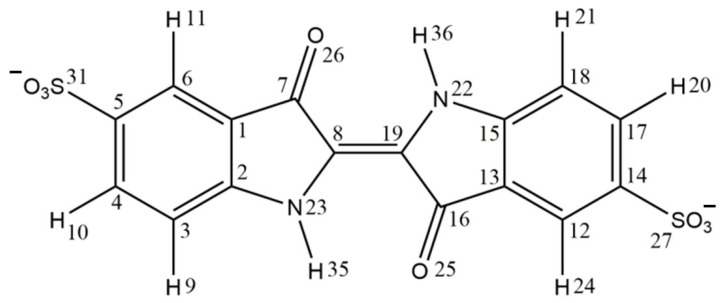
Dianionic indigo carmine (IC^2−^) and numbering scheme used in the discussion of results.

Indigo carmine, the sodium salt of 5,5′-indigodisulfonic acid (Scheme 1, IC^2−^), is a water-soluble derivative of indigo obtained by introducing sulfonate groups into positions 5 and 5′ of the phenyl rings of indigo. Its structure preserves the indigo chromophore, responsible for the characteristic blue color of these two dyes. IC shows great chemical versatility, receiving practical uses in many different areas, such as in analytical chemistry as a redox indicator [1,2], in medicine as a diagnostic tool, in the food industry as a coloring additive (E132) [3], in the pharmaceutical industry as a colorant for drugs and food supplements and in the textile industry to color blue jeans and other blue denim [4].

The IC dye has been considered a highly toxic indigoid [5]. It has been reported that contact with human skin can cause irritation and contact with eyes can cause permanent damage to the cornea and conjunctiva. In 1978, it was reported that ingestion of this synthetic dye could even be fatal, and it was indicated that it would lead to reproductive, neurological, developmental and acute toxicity [5]. When administered intravenously for medical procedures, such as in diagnostic methods and surgeries, it has been shown to be related to severe hypertension and cardiovascular and respiratory effects in patients. However, the exact causes of these side effects were not identified, and IC has been considered to be a useful and safe diagnostic tool [6,7]. The reported isolated cases of adverse effects to the use of IC were attributed to individual patient predispositions, rather than dye toxicity at the applied doses [8]. In 2023, the European Food Safety Authority (EFSA) issued an updated scientific opinion on the toxicity of IC [9]. The panel on Food Additives and Nutrient sources (ANS) confirmed the previously recommended acceptable daily intake of 5 mg/kg body weight per day for IC (E132) and noted that an intake below this level does not raise concerns regarding genotoxicity or subacute, chronic, reproductive or developmental toxicity. It was also noted that no population group exceeds the maximum recommended values of exposure. However, the EFSA panel also noted that infants, toddlers and children may be exposed to levels of IC above the acceptable daily intake if they consume more of the food products containing IC than the average population. This is in fact a real risk since IC is frequently used to make food products for young children, such as chewing gum, cereal, frozen desserts and toppings, more attractive.

While food dyes can potentially interfere with many biological processes and metabolites [10,11,12,13], a major concern considering the consumption of food dyes is their interaction with metal ions. Although synthetic dyes are considered safe for food purposes, many of them have the structural characteristics necessary for complexation with heavy metals. Many of these elements, such as iron, zinc, copper, nickel, etc., play essential roles in several biological functions and metabolic routes and therefore must be kept in the human body within restricted limits. Interactions between food dyes and metal ions can alter their bioavailability or produce complexes or other species with high toxicity. Therefore, thoroughly studying the interaction of food dyes with transition metal ions is very important in the context of the evaluation of their toxicity.

In recent years, the complexation of IC with various metal ions, such as copper(II), zinc(II), nickel(II), cobalt(II) or iron(II), has been investigated. However, a comparison of the literature reveals that, for some of these metal ions, there is no consensus regarding the number or stoichiometry of the complexes formed. Additionally, these studies lack detailed information on the structures of the complexes, which is essential for a deep understanding of the mechanisms of toxicity of IC. A twenty-year-old study by Salas-Peregrin and coworkers explored the interaction between copper(II) nitrate and IC, by using infrared spectroscopy, elemental analysis, magnetic measurements and thermal techniques. A solid salt, characterized as a 1:1 (metal:ligand) complex, was isolated from aqueous solution, but no information was provided regarding the sites of interaction between the metal and the ligand [14]. In a more recent spectrophotometric study by Zanoni et al. [15], the complexation between IC and several metal ions in aqueous solution was investigated. The authors proposed the formation of stable complexes between the IC ligand and the Cu(II), Ni(II), Co(II) and Zn(II) ions at pH 10, suggesting a 2:1 (metal:ligand) stoichiometry for all the complexes. It was concluded that the complexation with Zn(II) was significantly weaker, while the complex with Cu(II) appeared to be the most stable [15]. The interaction of IC with the chloride salts of Fe(II), Ni(II) and Cu(II) in aqueous solution was also studied by Haleim and coworkers [16] using UV/Vis absorption spectroscopy. The authors suggested the formation of complexes with 1:2 (metal:ligand) stoichiometry with Fe(II) at pH 9.4, Ni(II) at pH 7.2 and Cu(II) at pH 5.2. An additional complex with copper was identified, with 1:1 (metal:ligand) stoichiometry, in the pH range 6.8–8.8. The different results obtained by Zanoni [15] and Haleim [16] regarding pH conditions, number of complexes and their stoichiometries for Cu(II) and Ni(II) may be due to differences in the reagents used. In the study by Haleim et al., the metals were used in the form of chloride salts, while the study by Zanoni et al. did not disclose what form the metals were in. In a more recent (2017) study [17], Tavallali and coworkers proposed that the complexation of IC with Cu(II) occurs in a 2:1 stoichiometry (metal:ligand), in the pH range 7.5–10. That study used UV/Vis absorption spectroscopy and presented conclusions about the structure of the complexes and coordination sites, suggesting that the coordination of the metal to the ligand occurs through the N-H and C=O groups [17]. However, contrary to the older study mentioned above, the study by Tavallali et al. did not use H_2_O as a solvent but rather a mixture of solvents, H_2_O/DMSO (4:1 *v*/*v*). The use of different solvents can promote the formation of different species.

The poor agreement between the different reports on IC complexation with metal ions in water presented hitherto and the lack of structural details make a more complete and systematic characterization of these systems necessary. The reported higher stability of the copper(II) complexes compared to those formed by IC with other metal ions under physiological pH conditions motivated our interest in investigating this system using a variety of techniques, in order to achieve a comprehensive characterization of the Cu(II)/IC system in aqueous solution and in the solid state. NMR, infrared and UV/Vis spectroscopic studies were carried out to determine the number, stoichiometries and coordination sites of the complexes in this system. These experimental studies were complemented with Density Functional Theory (DFT) investigations (including time-dependent DFT calculations) that provided additional structural details.

## 2. Results and Discussion

### 2.1. Structure and Energetics of the Indigo Carmine Molecule

**Figure 1:**
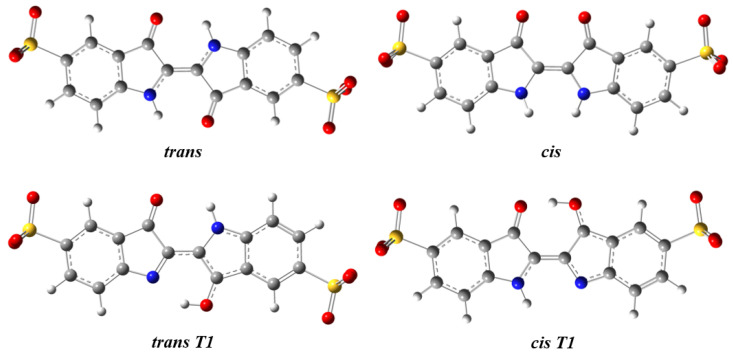
B3LYP/6-311++G(d,p) optimized geometries of the most stable forms of indigo carmine in H_2_O.

The indigo carmine molecule is composed of two 5-sulfo-3-indolinone fragments bound through a C=C double bond between the carbon atoms at positions 2 and 2′. Taking into account that the sulfonate substituents introduced at positions 5 and 5′ of the benzene rings ionize at a very low pH and that the pK_a_ of the amine groups is very high (pK_a_ > 11) [18,19], we can conclude that the IC^2−^ anion (Scheme 1) is the dominant form of indigo carmine in the pH region considered in this work. Two isomers can be expected for the IC molecule: the *trans* and *cis* isomers, along with several tautomers resulting from the transfer of hydrogen atoms between the carbonyl and amine groups. In this study, to characterize the structure of the IC^2−^ ligand in aqueous solution, the structures of its possible isomers and tautomers were optimized using the DFT B3LYP/6-311++G(d,p) level of theory, taking into account the bulk solvent effects of water. Figure 1 and Table 1 present, respectively, the optimized structures of the most stable forms of IC2^−^ in water and their relative Gibbs energies, symmetries and equilibrium populations at 298.15 K.

The equilibrium populations were estimated from the calculated relative Gibbs energies using the Boltzmann equation. Figure S1 and Table S1 in the supplementary material provide data on the additional higher-energy tautomers.

The obtained results allow us to conclude that the strongly dominant form of IC^2−^ in solution, with a population of ~100% at 298.15 K, is the *trans* structure, with C*_i_* point group symmetry. In this isomer, the indigo chromophore has intramolecular hydrogen bonds between the amine and carbonyl groups, which stabilize this form relatively to the *cis* form (in which these hydrogen bonds are absent) by 37 kJ/mol. According to the performed DFT calculations, the longest C-C bonds in the structure of the *trans* isomer are the C7-C8 and the C1-C7 bonds, with bond lengths of 1.494 and 1.464 Å, respectively. The remaining C-C bonds vary from 1.361 to 1.412 Å. This structural feature appears to be a consequence of the charge delocalization that occurs with the establishment of the intramolecular interactions between the amine and the neighboring carbonyl groups and the formation of the 6-atom *pseudo*-rings they originate. *Trans* indigo carmine presents a planar skeleton, while the optimized structure of the *cis* isomer has a substantial twist between the planes of the rings around the C=C central double bond (C7-C8=C19-C16 dihedral angle of 11.4°). The energy barrier for the *trans* → *cis* conformational isomerization was estimated as being 84 kJ/mol, according to the performed relaxed potential energy profile calculated at the DFT B3LYP/6-311++G(d,p) theoretical level (Figure S2). Therefore, this process is not expected to occur at room temperature without an external source of energy. The remaining tautomers of indigo carmine have relative energies considerably higher than that of the *trans* form, so they can be concluded to have negligible populations (~0%) in the relevant experimental conditions. The *trans* isomer will be, therefore, the only species considered in this study for the DFT calculation of the NMR, IR and UV/Vis spectra of the free ligand, which are discussed in the following sections and support the interpretation of the experimental results.

According to the performed literature search, no X-ray structure of indigo carmine has yet been published. Therefore, we present in Table 2 selected conformationally relevant structural parameters of the DFT optimized structure of the *trans* form.

### 2.2. Cu(II) Complexes of Indigo Carmine

**Figure 2:**
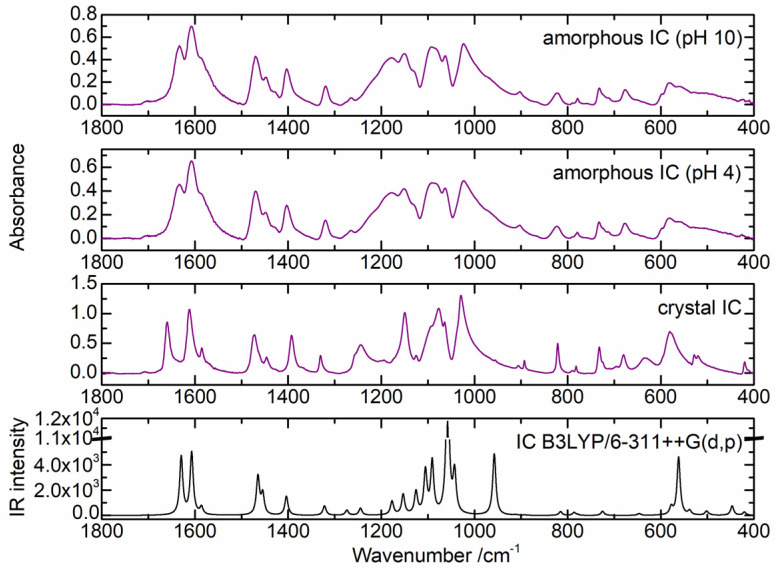
ATR-FTIR spectra (1800–400 cm^−1^) of the crystalline IC solid (**middle bottom**) and the powder samples obtained after evaporation of the solvent of 0.05 mol dm^−3^ IC solutions at pH = 10 and 4 (**top** and **middle top**). The B3LYP/6-311++G(d,p) calculated spectrum of IC^2−^ *trans* is also included (**bottom**).

In a preliminary step, before considering complexation with Cu^2+^ ions, the infrared spectrum of the IC ligand in its commercial crystalline form was obtained and analyzed based on the DFT calculated spectrum simulated of the IC^2−^ anion. Additionally, aqueous solutions of IC were prepared at different pH values and were left at room temperature until complete evaporation of the solvent. The blue powder obtained after evaporation of the solvent of each sample was then also analyzed by ATR-FTIR. From the analysis of these spectra, it was possible to conclude that in the pH region between 4 and 10, the ATR-FTIR spectrum of IC remains unaltered. However, the spectra of the prepared powder samples present differences relative to the spectrum of the crystalline indigo carmine obtained commercially. Figure 2 shows the ATR-FTIR spectra of the IC samples obtained from solutions at pH 4 and pH 10, along with the spectrum of the crystalline IC sample. These are compared with the IR spectrum calculated for the IC^2−^ anion at the B3LYP/6-311++G(d,p) level of theory, taking into account the bulk solvent effects of water. The vibrational frequencies of the theoretical spectrum were multiplied by an optimized scaling factor (0.984) which was determined by a linear fit between selected experimental vibrational frequencies of the crystalline IC and the corresponding theoretical frequencies (Figure S3).

Comparing the spectra obtained for the powder and the crystalline IC samples, it is possible to observe the expected significant broadening of the bands in the spectra of the powder samples. Furthermore, some deviations in the frequencies of the bands are also observed, namely the band due to the carbonyl stretching vibration, which appears at 1659 cm^−1^ in the crystalline IC spectrum, appears shifted to a lower frequency in the spectra of the powder samples (1635 cm^−1^). Differential Scanning Calorimetry (DSC) was used to confirm that there was no water present in the solid IC powder samples, and therefore, the observed changes were attributed to a loss of the crystalline arrangement of the IC molecules and formation of an amorphous phase. The solid samples of the Cu(II) complexes used in the ATR-FTIR studies were prepared following the same procedure presented for the ligand, and therefore, the experimental spectrum of the IC amorphous phase will be considered for comparison with the samples of complexes.

**Figure 3:**
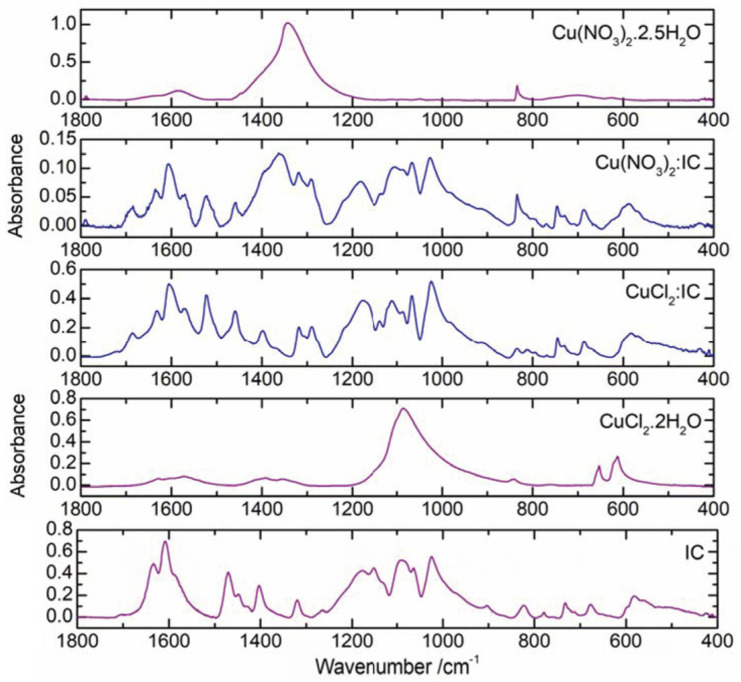
ATR-FTIR spectra (1800–400 cm^−1^) of the solid samples obtained from aqueous solutions of Cu(NO_3_)_2_·2.5H_2_O at pH 8.17, Cu(NO_3_)_2_·2.5H_2_O:IC 0.010:0.005 mol dm^−3^ at pH 8.13, CuCl_2_·2H_2_O:IC 0.010:0.010 mol dm^−3^ at pH 9.95, CuCl_2_·2H_2_O at pH 10.11 and IC at pH 8.07 (**top** to **bottom**). Note: the spectra of the salts may include bands from Cu(II) hydroxide that may have formed at the pH values considered.

Published studies have reported complexation between IC and copper(II) nitrate [14,17] or copper(II) chloride [17]. It is possible that the use of different salts may be one of the reasons for the lack of agreement between the structures proposed for the complexes in those studies, since different copper salts can promote the formation of different complexes. To analyze this effect, we compared the complexes obtained with both salts in the solid state. Figure 3 shows the ATR-FTIR spectra of the solid samples obtained from aqueous solutions of Cu(NO_3_)_2_·2.5H_2_O, CuCl_2_·2H_2_O, IC and solutions of the complex(es) formed between these salts and IC.

It can be noted that the spectra of the metal:ligand samples suggest, for both salts, formation of the same type of complex(es), since the new bands detected in comparison to the ligand spectrum are the same in both spectra. It seems that, at least for the complex(es) formed in the solid state, both the nitrate and chloride metal salts originate the same type of complexes. The two spectra of the metal:ligand samples are very similar, except for the region between 1250 and 1500 cm^−1^, in which the Cu(NO_3_)_2_:IC spectrum presents a relatively wide band that impairs its analysis. This very broad band appears to have contributions of vibrations from the nitrate groups present in the sample (originating from the reactant Cu(NO_3_)_2_·2.5H_2_O), since a very intense absorption band is observed at 1343 cm^−1^ in the spectrum of this salt. For this reason, the CuCl_2_·2H_2_O salt was chosen to carry out the subsequent experiments. It should be noted that at these high pH values (8–10), it is probable that the spectra of the salts show additional bands due to Cu(II) hydroxide. 

**Figure 4:**
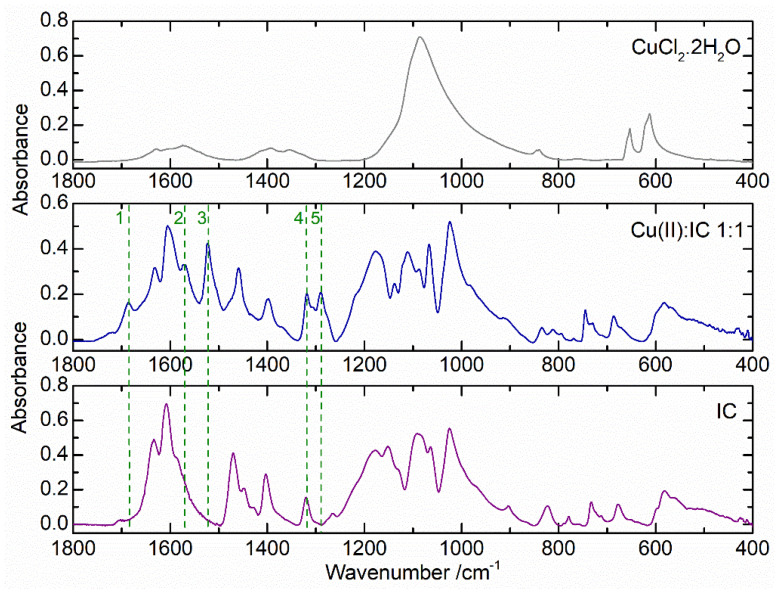
ATR-FTIR spectra (1800–400 cm^−1^) of the solid samples obtained from aqueous solutions of CuCl_2_·2H_2_O at pH 10.11 (**top**), Cu(II):IC 0.010:0.010 mol dm^−3^ at pH 9.95 (**middle**) and IC at pH 9.95 (**bottom**).

The spectrum of the Cu(II):IC sample obtained from a 0.010:0.010 mol dm^−3^ solution at pH 9.95 (Figure 4, middle) presents significant differences compared to the spectra of the reagents, indicating that complex formation has occurred. This is revealed by the appearance of new bands at 1685 (band 1), 1522 (band 3) and 1288 cm^−1^ (band 5) and the increase in the intensity of the bands at 1572 (band 2) and 1321 cm^−1^ (band 4) relative to the spectra of the reagents. The region of the spectrum between 3700 and 2700 cm^−1^ is dominated by an intense band due to the symmetric and anti-symmetric stretching vibrations of the H_2_O molecules possibly coordinated in the complexes, together with N-H and C-H stretching vibrations (Figure S4). 

In addition to the conditions already mentioned (Figure 3 and Figure 4), in the study of the Cu(II):IC system by ATR-FTIR, the molar ratios 1:1, 1:2 and 2:1 (M:L) were also considered, with solutions at pH 4, 6, 7, 8 and 10. It was found that for all the conditions analyzed, the same complex was isolated in the solid state since, despite some slight differences in the relative intensities of the bands, all spectra present a similar profile, irrespective of the sample (Figure S5). For the various molar ratios, 1:1, 1:2 and 2:1, pH 8–10 appears to be the most favorable for complexation, since under these conditions, the intensity of the new absorption bands is maximum. Comparing the relative intensities of the bands in the spectra obtained for the samples with various molar ratios and the same pH (pH 8, Figure S5), the presence of a larger relative amount of the complex in the sample with a molar ratio of 1:2 (M:L), in which it is expected that a 1:2 stoichiometry complex is favored, can be concluded. This may suggest that this is the stoichiometry of the complex formed between Cu(II) and IC under the experimental conditions used in the present study.

**Figure 5:**
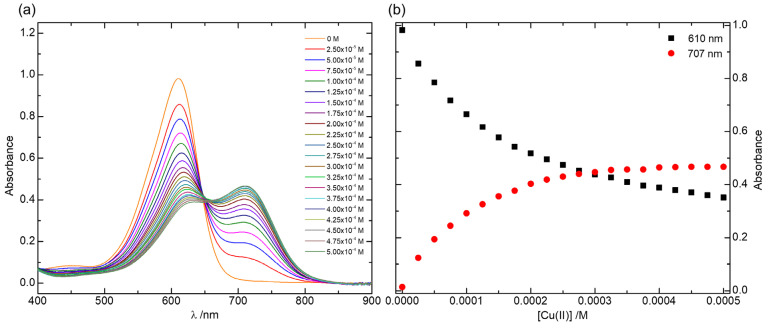
(**a**) Absorption spectra of an IC aqueous solution (5 × 10^−5^ mol dm^−3^) in the presence of increasing concentration of Cu(II) (steps of 2.5 × 10^−5^ mol dm^−3^) at pH 6.2; (**b**) absorbance intensity at 610 nm (black squares) and 707 nm (red circles) as a function of Cu(II) concentration at pH 6.2.

A previously published [17] study on the complexation behavior of IC with Cu(II) reports FTIR data on this system. The IC+Cu(II) spectrum presented in that work is, however, very different from the ones we obtained for our samples; namely, the new bands 1 and 3, attributed in our work to the Cu(II):IC complex, are not present in the spectrum shown in that publication. However, in that previous study, a mixture of H_2_O:DMSO 4:1 was used as a solvent, so it is plausible to admit that the authors may have observed a Cu(II):IC complex different from the one we obtained in our work. In fact, in that publication, the complex found was characterized as a 2:1 (metal:ligand) species, contrary to our results which seem to point to a 1:2 complex. To obtain additional information on the stoichiometry and determine the structure of the Cu(II):IC complex found in the present work, we carried out additional experiments. Specifically, we studied the complexation between IC and Cu(II) in aqueous solution by UV/Vis absorption spectroscopy. This study was carried out at pH 6.2, since a preliminary study (Figure S6) showed that in solutions with a large excess of metal (10:1), there is formation of Cu(II) hydroxide above pH 7. UV/Vis absorption spectra were then obtained for an aqueous solution of IC with increasing concentrations of Cu(II) (metal:ligand molar ratio in the range 1:2 to 10:1) at pH 6.2 (Figure 5a).

Figure 5a,b clearly show that, as the concentration of Cu(II) increases, there is a gradual decrease in the intensity of the free IC absorption band (610 nm), with a simultaneous increase in the absorption intensity at 707 nm, which corresponds to the absorption maximum of the new species formed as IC is consumed. There is no well-defined isosbestic point, so it is possible that competition between several equilibria for the formation of different Cu(II)/IC complexes is occurring.

**Figure 6:**
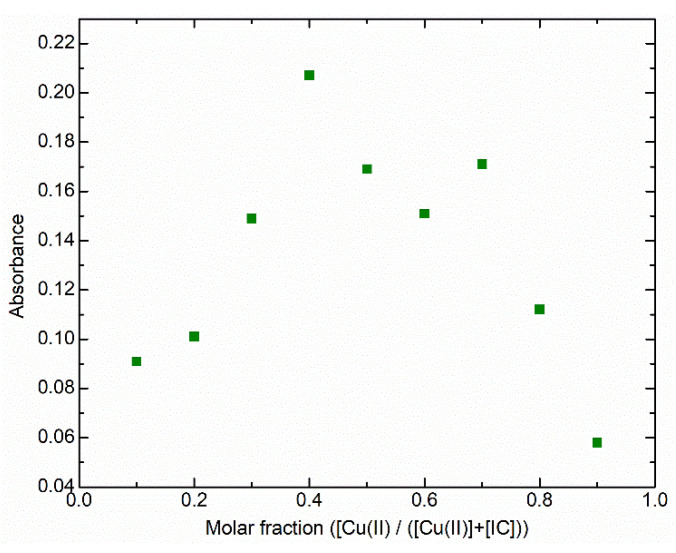
Job’s plot showing the UV/Vis absorbance at 707 nm as a function of the molar fraction [Cu(II)]/([Cu(II)] + [IC]) for aqueous solutions with total concentration ([Cu(II)] + [IC]) 1 × 10^−4^ mol dm^−3^, at pH 6.2.

In an attempt to determine the stoichiometry of the complex(es) formed, Job’s method was used. This method has, however, certain limitations that Job pointed out in his early work [20], such as that it would only be applicable when there is a single complex in solution. Later studies have shown that the concentration of the A_a_B_b_ complex is still a maximum in the stoichiometric a/b ratio if the concentrations of the competing complexes are much lower for this ratio [21,22]. The Job’s plot (Figure 6) shows two absorption maxima close to molar fractions 0.4 and 0.7 (the spectra were repeated multiple times with different solutions to rule out errors), suggesting that there will be more than one complex in solution, making it impossible to draw conclusions about their exact stoichiometries due to the method’s limitations. Nonetheless, these maxima suggest that one of the two complexes will have a higher proportion of the ligand, close to 1:2 (maximum at 0.4), and the other complex will have a higher proportion of the metal, close to 2:1 (maximum at 0.7). This result is very relevant, since it reveals the formation of more than one complex in agreement with the previous finding of a poorly defined isosbestic point (Figure 5a).

**Figure 7:**
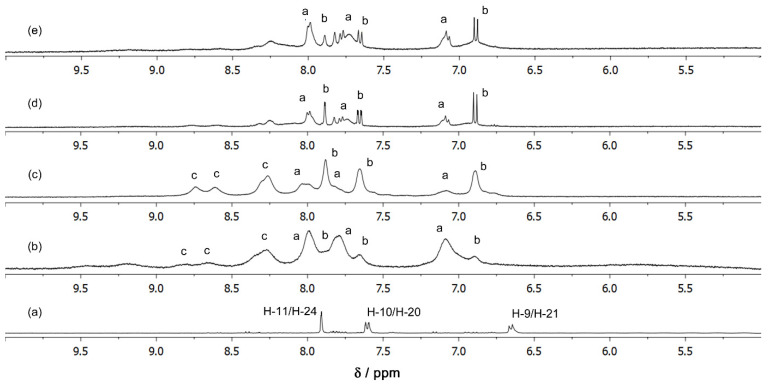
^1^H NMR spectra of H_2_O:D_2_O solutions of (**a**) IC 5.0 mmol dm^−3^, pH* 8.05; (**b**) CuCl_2_:IC 2.5:5.0 mmol dm^−3^, pH* 5.95; (**c**) CuCl_2_:IC 5.0:5.0 mmol dm^−3^, pH* 8.10; (**d**) Cu(NO_3_)_2_:IC 2.5:5.0 mmol dm^−3^, pH* 8.04; (**e**) CuCl_2_:IC 2.5:5.0 mmol dm^−3^, pH* 8.10 (pH* is used to indicate the pH value measured in solutions with a 70%:30% mixture of H_2_O/D_2_O as solvent, after standardization with aqueous (H_2_O) buffers; see Section 3.1 for details). Temperature = 298.15 K.

To further investigate the Cu(II):IC system in aqueous solution, ^1^H NMR spectra were obtained for IC solutions in the absence and in the presence of the Cu^2+^ metal ion (Figure 7 and Figure S7), for different molar ratios and pH values and with two different copper(II) salts, Cu(NO_3_)_2_ and CuCl_2_ (the full assignment of the ligand ^13^C and ^1^H spectra is also shown in Figures S8–S10). The spectra reveal almost the same complexation pattern with both salts for the same pH and molar ratio values (cf. Figure 7d,e). Despite the complexity of the system and the broad signals observed, by comparing the intensities of the signals in the spectra of the various solutions, it was possible to identify the major complexes, **a**, **b** and **c**, and establish that they are most probably species with a (metal:ligand) stoichiometry of 1:2, 2:1 and 1:1, respectively.

Due to the rapid nuclear relaxation induced by the paramagnetic properties of the Cu^2+^ (3*d*^9^) metal ion, the ^1^H NMR signals of the complexed ligands show very broad signals and/or shifts to high frequencies when compared to the signals of the free ligand, making it difficult to unequivocally assign some of the signals. The most probable assignment of the ^1^H NMR signals of the complexes is shown in Table 3. 

The shifts resulting from the binding of the ligand to paramagnetic metals can result from the direct delocalization of the spin density (contact shift) or from the dipolar interactions in space (pseudo-contact shift) of the unpaired electrons of the metal. The Cu^2+^ (3d^9^) metal ion in the complexes has contributions that reflect both the contact shift and the pseudo-contact shift, resulting in the observation of very broad ^1^H NMR signals, in particular for mononuclear complexes. These results align with the comparable effects observed in earlier studies on the paramagnetic 8-hydroxyquinoline complex of Cr(III) [23] and other mononuclear complexes of the Cu(II) metal ion [24,25]. On the other hand, binuclear copper(II) complexes exhibit narrower ^1^H NMR signals, usually with signal widths two orders of magnitude smaller than mononuclear analogs. However, the broadening of the signals in mono- and binuclear complexes generally increases with an increase in the concentration of free metal in the solution, as shown in Figure S7. The broad signals observed for complexes **a** and **c** support the hypothesis that the complexes should be mononuclear species of 1:1 and 1:2 (metal:ligand) stoichiometries, CuIC and Cu(IC)_2_, respectively, while species **b** should correspond to a binuclear species of 2:1 (metal:ligand) stoichiometry, Cu_2_IC. 

**Figure 8:**
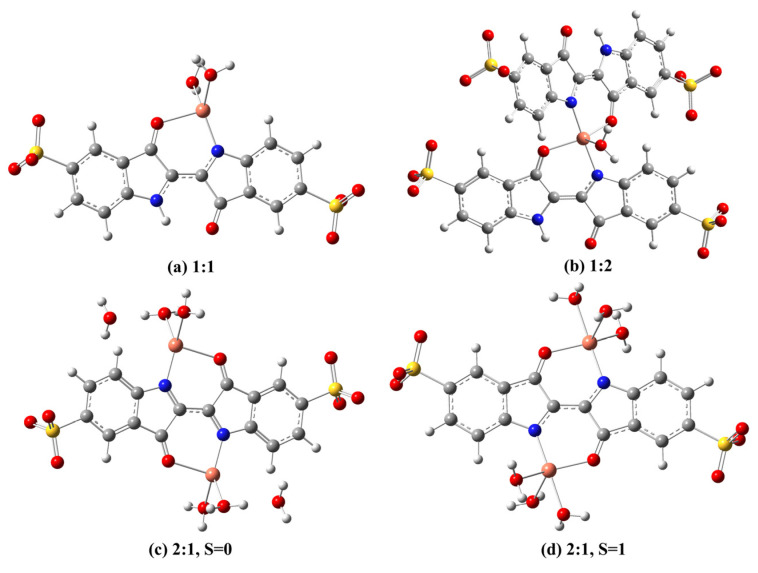
Optimized geometries of the (**a**) 1:1 and (**b**) 1:2 Cu(II)/IC complexes; two possible structures for the 2:1 Cu(II)/IC complex, (**c**) considering total spin as 0, singlet state, and (**d**) considering total spin as 1, triplet state (optimized at the B3LYP/LanL2DZ/6-311++G(d,p), in water).

Using the structural information obtained from the FTIR, UV/Vis and NMR experiments as input information, DFT calculations were carried out with the aim of characterizing the structures of the complexes in greater detail. To determine their lowest-energy most probable geometries, the various possible isomeric structures with 1:1, 1:2 and 2:1 Cu(II):IC stoichiometries were considered. It is expected that coordination of IC to the metal ion occurs through the carbonyl O and the deprotonated N atoms since, although the pK_a_ value of IC is relatively high (pK_a_ > 11) [18,19], deprotonation can occur at lower pH values in the presence of metals that can form stable complexes with IC (an assumption corroborated by the evidence of increase in the extent of complexation with increasing pH). Taking into account that the usual coordination numbers for Cu(II) complexes with *N-* and *O-*donor ligands in aqueous solution are six, five and four (in fact, two of the relevant aqua species of Cu(II) are the [Cu(OH_2_)_6_]^2+^ and [Cu(OH_2_)_4_]^2+^ ions), six-coordinated metal centers were considered for all the input structures, by including coordinated H_2_O molecules in the remaining positions of the first coordination sphere of Cu(II). The optimization of the structures was carried out at the B3LYP/LanL2DZ/6-311++G(d,p) theoretical level, taking into account the bulk solvent effects of water. For all the structures, the optimization procedure converged to geometries in which one or two H_2_O molecules were expelled from the first coordination sphere and remained hydrogen bonded to different positions in the IC ligand. In a subsequent step, these H_2_O molecules were removed, and the structures were reoptimized. The optimized structures are shown in Figure 8 and reveal that Cu(II) in these complexes shows preference for metal centers with coordination numbers 4 and 5 (in the 2:1 singlet structure shown in Figure 8, one H-bonded water molecule was left in each center to allow comparison of energy with the triplet isomer).

The **1:1** complex has a metal center with coordination number 4 in a distorted tetrahedral arrangement, global charge −1 and C_1_ point group symmetry. The **1:2** structure has global charge −4, C_1_ point group symmetry and coordination number 5 in a metal center with a square pyramidal geometry. It is not unexpected to find different coordination numbers and geometries for Cu(II) complexes. Indeed, due to the non-spherical symmetry of the *d*^9^ configuration and the Jahn–Teller effect on six-coordinate geometries, the coordination sphere of Cu(II) ions is characterized by its flexibility, with non-rigid geometries (fluxional behavior) that include regular octahedral, elongated tetragonal octahedral, square planar, tetrahedral, trigonal bipyramidal and square pyramidal metal centers [26,27,28].

The **2:1** stoichiometry structure has two Cu(II) ions, and therefore, two possibilities for the global spin of the complex were considered, namely the singlet state, assuming that pairing of the unpaired electrons of the two metal ions (of 3*d*^9^ configuration) occurs, and the triplet state, assuming that in the structure, there are two unpaired electrons (that is, assuming ferromagnetic coupling between the metal ions). Both structures were optimized and converged to slightly different geometries. The **2:1** singlet structure converged to a geometry with coordination number 4, in which two water molecules were expelled from the first coordination sphere of each metal center, and has C*_i_* point group symmetry. The **2:1** triplet structure converged to a geometry with coordination number 5, square pyramidal metal centers and C*_i_* point group symmetry. The 2:1 singlet configuration was reoptimized using the five-coordinated triplet structure as a starting geometry, and again the fifth water molecule was expelled from each metal center. Comparison of the energies of the singlet and triplet isomeric 2:1 structures reveals that the singlet is more stable than the triplet by 61.8 kJ/mol, and therefore, these results suggest the singlet configuration for the 2:1 structure. This result is in complete agreement with the narrow NMR signals observed for complex **b** (Cu_2_IC), in contrast with the broader NMR signals observed for the mononuclear complexes **a** and **c**. These results also show that antiferromagnetic coupling occurs between the two metal centers of the 2:1 complex observed in aqueous solution. Through-ligand antiferromagnetic coupling has also been observed for other Cu(II) dinuclear complexes with long intermetallic distances (e.g., distances of 5.7 [29], 11.3 [30] and 12.3 [31] angstroms). According to our DFT calculations, the distance between the two metal ions in the 2:1 Cu(II)/IC complex is 6.24 angstroms, which is comparable to those examples in the literature. From a fundamental perspective, this is also an interesting system since it allowed us to investigate through-ligand exchange magnetic coupling by using NMR spectroscopy coupled with DFT calculations.

**Figure 9:**
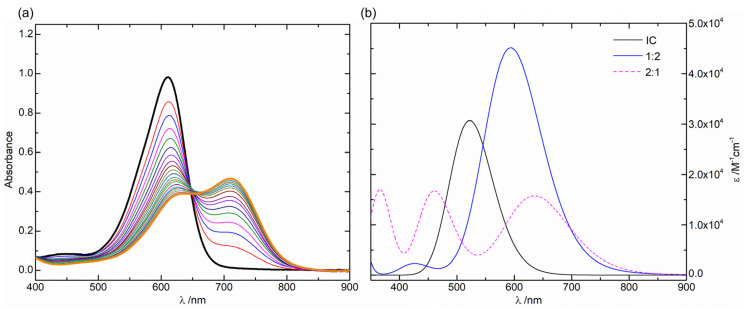
(**a**) Absorption spectra of an IC aqueous solution (5 × 10^−5^ mol dm^−3^) in the presence of increasing concentration of Cu(II) (steps of 2.5 × 10^−5^ mol dm^−3^) at pH 6.2 (black trace: [Cu(II)] = 0 mol dm^−3^); (**b**) TD-DFT UV/Vis simulated spectra (CAM-B3LYP/LanL2DZ/6-311++G(d,p), in water) of IC *trans* and the 1:2 and 2:1 Cu(II):IC structures.

Once plausible structures for the complexes have been determined, one is now ready to analyze the obtained UV/Vis absorption and FTIR results in more detail. The UV/Vis absorption spectra were simulated by TD-DFT for the 1:1, 1:2 and 2:1 singlet structures presented in Figure 8 and for the free ligand (Figure 9b). TD-DFT calculations on molecules that have an open shell ground state can generate excited states with unphysically large amounts of spin contamination. This difficulty can be partially overcome by only considering excited states that preserve <*S*^2^> within appropriate limits, neglecting states for which the difference between <*S*^2^> and its exact value goes beyond the acceptable error [32]. The spectrum simulated for the 1:1 structure will not be analyzed since for this structure, almost all the calculated excited states showed a high degree of spin contamination under the calculation conditions used. 

One can observe that the spectrum calculated for the **1:2** structure correctly reproduces the shift observed in the experimental spectrum of the Cu(II)/IC system at pH 6.2 in the region between 350 and 900 nm, in agreement with the NMR experimental results at pH 6 which indicate an equilibrium dominated by complex **a** (1:2 Cu:IC), with a significant presence of complex **c** (1:1). We should note that the relative intensities of the theoretical bands do not necessarily correlate with the experimental spectra, as these intensities depend on the experimental relative concentrations of IC and the complex. The spectrum calculated for the **2:1** structure presents an additional absorption band, at shorter wavelengths (ca. 454 nm), which is not observed experimentally, and therefore, this complex shall not be present in significant amounts at pH 6, also in accordance with the NMR results. The band observed at 710 nm for the **1:2** complex can be assigned based on the TD-DFT calculations to the contributions of two bands predicted at 592 and 615 nm, which involve transitions with π–π* character. The calculated vertical excitation energies, oscillator strengths, wavelengths and major contributions to the excited states of indigo carmine and its 1:2 and 2:1 complexes with Cu(II) are summarized in Table S2, Figures S11 and S12. 

EPR spectra were obtained for three different Cu(II):IC solutions, with varying pH and metal:ligand molar ratios. The spectra for solutions at pH 8 and molar ratios of 1:2 (2.5:5 mmol/dm^−3^) and 1:1 (10:10 mmol/dm^−3^) provided interesting results, suggesting the presence of two paramagnetic species (Figure S13). Considering that the NMR data obtained for similar pH and molar ratios (Figure 7c–e) indicate the coexistence of three Cu(II):IC complexes under these conditions, these results are in complete agreement with our proposal that two of these complexes are paramagnetic while one is diamagnetic. EPR spectra were also obtained for a 2.5:5 mmol/dm^−3^ solution at pH 6; however, in these conditions, the spectrum does not show a reasonable signal/noise ratio. The distribution of copper into multiple complexed species, along with the presence of free metal, probably contributed to the difficulty in obtaining a good spectrum.

**Figure 10:**
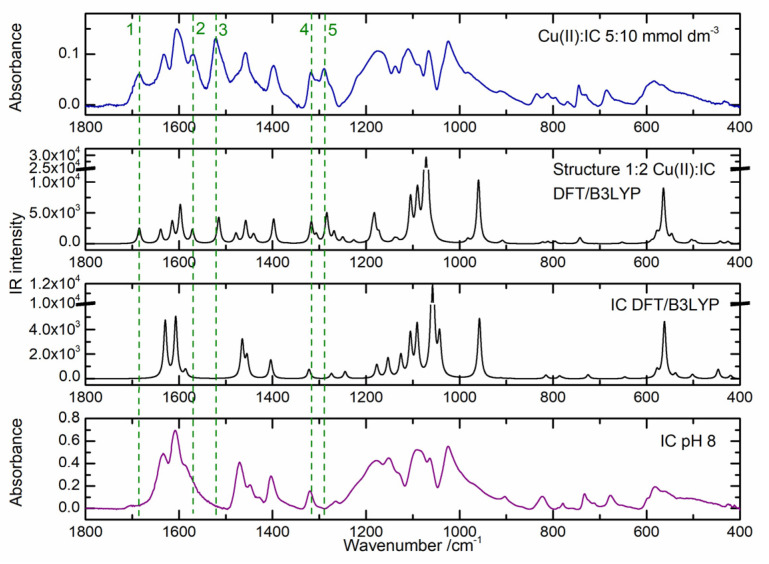
ATR-FTIR spectra (1800–400 cm^−1^) of the solid powder samples obtained from an IC aqueous solution (**bottom**) and from a Cu(II):IC 5:10 mmol dm^−3^ aqueous solution (**top**), both at pH 8, in comparison with the DFT/B3LYP calculated spectra for the *trans* IC conformer (**middle bottom**) and the **1:2** Cu(II):IC structure (**middle top**). The vibrational frequencies of the theoretical spectra were scaled by 0.984 and 0.986, respectively, for the ligand and complex spectra (Figures S3 and S15).

The theoretical infrared spectra were also calculated for the same structures in order to structurally characterize the species observed in the solid state (Figure S14). Comparing the theoretical spectra with the experimental one, it can be seen that the spectra of the **2:1** (both singlet and triplet) structures fail to predict the new bands attributed to the complex, so these two structures do not seem to correspond to the observed complex. On the other hand, the spectra calculated for the **1:1** (complex **c**) and **1:2** (complex **a**) structures reproduce the experimental spectrum much better, and the agreement is almost perfect for the spectrum calculated for the **1:2** structure, which correctly predicts not only the vibrational frequencies of new bands of the complex but also their relative intensities (Figure 10).

As indicated previously, the most significant changes observed in the experimental spectrum of the solid Cu(II):IC sample relative to the experimental IC spectrum are the presence of five new bands, at 1685, 1572, 1522, 1321 and 1288 cm^−1^. In perfect agreement with this, these bands are absent in the theoretical IC spectrum and are predicted for the 1:2 complex at 1686, 1572, 1516/1515, 1318 and 1285 cm^−1^, respectively. The good agreement between the calculated spectrum for the **1:2** structure and the experimental spectrum obtained for the solid sample of Cu(II):IC allows us to propose the **1:2** structure in Figure 8 as having the essential structural features of the complex formed in the solid state. Based on this good agreement, we can assign the bands observed for the complex with a high degree of confidence. The new bands 1 and 3 are attributed to the stretching vibrational mode of the carbonyl groups. Band 1, predicted at 1686 cm^−1^, corresponds to the stretching vibration of the C=O bond of the free carbonyl groups, coupled with the C=C stretching of the double bond at position 2, and band 3, predicted at 1516/1515 cm^−1^, corresponds to the C=O stretching vibration of the coordinated carbonyl groups, conjugated with C=C stretching and N-H in-plane angular deformations. These results are particularly relevant as they confirm the coordination of only one carbonyl group of each ligand molecule to the metal. The coordination of the carbonyl O atom involves donation of electrons from the ligand to the metal, making the C=O bond weaker and therefore changing the stretching vibrational frequency of the coordinated C=O groups to a lower frequency relative to the uncoordinated C=O group. Band 2, predicted at 1572 cm^−1^, is due to C-C stretching vibrations in the phenyl rings. Band 4, predicted at 1318 cm^−1^, involves mainly C-C stretching vibrations of the phenyl rings, but it also has some contribution of C=O stretching of the coordinated carbonyl groups. Finally, band 5, predicted at 1285 cm^−1^, is due to N-C stretching coupled with in-plane C-H angular deformation. The EDS results (Table 4) indicate, however, that the actual structure of the complex formed in the solid state has a higher percentage of copper than the one predicted for the 1:2 structure. Bearing in mind that in the solid state, a rearrangement of the 1:2 structure may occur, such that polymeric structures based on the 1:2 repeating unit may form, we carried out additional DFT calculations for the smallest oligomer of this type, a structure with 2:3 Cu(II):IC stoichiometry, to predict its stability and vibrational spectrum. Such a structure was found to be indeed stable, and its vibrational spectrum was also found to correctly reproduce the experimental infrared spectrum of the solid sample (Figures S16 and S17). For this structure, the agreement between the experimental and the expected copper percentage is better and will improve for longer oligomers. Therefore, these results suggest that the solid-state structure of the complex will likely consist of a polymeric arrangement involving repetition of the 1:2 complex unit.

## 3. Materials and Methods

### 3.1. Materials and Preparation of Samples

Commercially available disodium 2-(3-hydroxy-5-sulfonato-1*H*-indol-2-yl)-3-oxoindole-5-sulfonate (indigo carmine, IC, Merck, Darmstadt, Germany), copper(II) nitrate hemi(pentahydrate) (Sigma-Aldrich, St. Louis, MO, USA) and copper(II) chloride dihydrate (Merck) were used as received. For the UV/Vis, EPR and ATR-FTIR experiments, solutions were prepared in Milli-Q H_2_O, and the pH was adjusted by the addition of HClO_4_ and NaOH solutions. The samples were kept in the dark until used. Powder samples for the ATR-FTIR experiments were prepared by evaporating the solvent at room temperature from the solution samples. The sample for the EDS analysis was obtained by filtration of the precipitate formed in a 10:10 mmol dm^−3^ Cu(II):IC solution after being kept at 5⁰C for a week. After filtration, the precipitate was dried in a desiccator. The solutions for the NMR experiments were prepared in a 70%:30% mixture of H_2_O/D_2_O, and the pH was adjusted by adding DCl and NaOD; the reported pH* values are the direct readings from the pH-meter at room temperature, after standardization with aqueous (H_2_O) buffers. ^1^H NMR (D_2_O, 500 MHz, δ (ppm), Free IC: 7.90 (s, 2H); 7.58 (d, 2H); 6.66 (d, 2H); Complex **a**: 7.98 (broad, 2H); 7.80 (broad, 2H); 7.08 (broad, 2H); Complex **b**: 7.89 (s, 2H); 7.64 (d, 2H); 6.90 (s, 2H); Complex **c**: 8.84 (broad, 2H); 8.67 (broad, 2H); 8.26 (broad, 2H). 

### 3.2. Instrumentation

The ATR-FTIR spectra were acquired in a Thermoscientific Fourier Transform Infrared Spectrometer—Nicolet iS5 iD7 ATR (Thermo Fisher Scientific, Waltham, MA, USA) (resolution 1 cm^−1^), using the OMNIC 8 program for spectra collection and analysis. The animation visualization module of the GaussView 6.0 program was used to facilitate the assignment of the vibrational modes. A Shimadzu spectrometer UV-2100 (Shimadzu, Tokyo, Japan) was used to obtain the UV/Vis absorption spectra, and the ^1^H and ^13^C NMR spectra were acquired in a Bruker Avance 500 NMR spectrometer. The HSQC and HMBC (2D NMR) spectra were recorded on the same spectrometer. In the NMR spectra, the methyl signal of *tert*-butyl alcohol was used as an internal reference (δ 1.2 for ^1^H and δ 31.2 for ^13^C) relative to TMS. The ^13^C spectra were obtained using Waltz-16 proton decoupling and taking advantage of the nuclear Overhauser effect (as a result, signal intensities for ^13^C spectra are not quantitative). The X-ray microanalysis (EDS) was conducted with the Bruker QUANTAX system which includes the Bruker Nano XFlash^®^ detector (Bruker, Billerica, MA, USA). The Bruker Nano XFlash^®^ detector is an energy-dispersive X-ray detector that works according to the principle of the silicon drift detector, with a 133 eV energy resolution (Mn Ka) @ 100 kcps. The detector has an effective area of 10 mm^2^ and is cooled by a Peltier element. The elements in the range B (Z = 5) to Am (Z = 95) can be identified and quantified. The software module uses a standardless PB-ZAF method for quantification. This system is installed in the TESCAN VEGA 3 SBH—Easy Probe SEM (TESCAN, Brno, Czechia). For the data analysis, the ESPRIT 1.9 Software was used. The EPR spectra (X-band, 0.34 T, 9.5 GHz) were recorded on a continuous-wave Bruker EMX spectrometer. Typical instrument settings were microwave power 0.6 mW, modulation amplitude 16.4 G, modulation frequency 100 kHz, sweepwidth 600.0 G and 256 scans for each spectrum. The solution samples were placed in a 3 mm EPR tube for the measurements.

### 3.3. Computational Details

The geometries of the conformers and tautomers of IC and its Cu(II) complexes were optimized by DFT, using the B3LYP [33] functional, the LanL2DZ [34,35] pseudopotential and the associated valence basis sets for Cu and the 6-311++G(d,p) [36] basis set for the remaining atoms. Vibrational frequency analysis was carried out to assess the nature of the stationary points; that is, the absence of imaginary frequencies allowed confirmation that these are minima on the potential energy surface. The potential energy profile for the *cis*-*trans* conformational interconversion in the most stable tautomer of indigo carmine was obtained by scanning the conformationally relevant dihedral angle (C7-C8=C19-C16) with optimization of the remaining structural parameters. 

The calculated harmonic vibrational frequencies were scaled with scaling factors designed to correct for limitations introduced by the incomplete basis set, the partial treatment of electronic correlation and vibrational anharmonicity [37,38]. These scaling factors were determined by linear fits between selected experimental vibrational frequencies of the studied samples and the corresponding theoretical frequencies. The theoretical infrared spectra depicted in the Figures were simulated by employing Lorentzian functions with a full width at half maximum (FWHM) of either 6 or 4 cm^−1^, centered at the scaled calculated vibrational frequencies.

The optimization of the structures was carried out accounting for the bulk solvent effects of water through the application of the IEFPCM (integral equation formalism variant of the polarizable continuum model) [39,40]. 

The UV/Vis absorption spectra of both the ligand and complexes were computed using the TD-DFT (Time-Dependent Density Functional Theory) method, using the long-range corrected hybrid CAM-B3LYP [41] functional and the basis sets indicated above. This functional, integrating Hartree–Fock exchange interaction in varying degrees depending on interelectronic distance, has demonstrated its ability to yield vertical excitation energies close to experimental values across a diverse range of molecules [42,43]. The DFT and TD-DFT calculations were conducted with the Gaussian 16 [44] code, while visualization of structures and molecular orbitals was enabled by the GaussView 6.0 software.

## 4. Conclusions

A full speciation study and structural characterization of the Cu(II):IC complexes was carried out in aqueous solution and in the solid state by using FTIR, UV/Vis absorption and NMR spectroscopies, complemented with DFT and TD-DFT calculations. These studies showed that three types of Cu(II)/IC complexes, with 1:1, 1:2 and 2:1 metal:ligand stoichiometries, are formed in aqueous solution and are favored in the pH range 6–7. These complexes are stable over time, and a polymeric arrangement involving repetition of the 1:2 complex unit was proposed to form in the solid state. The combination of these spectroscopic techniques with computational methods allowed the structures of the complexes formed to be characterized for the first time in appreciable detail. Information on their metal center coordination geometries, coordination sites of the ligand and full geometries are provided based on DFT calculations. The 1:1 and the 2:1 complexes show distorted tetrahedral metal centers, while the 1:2 complex has a square pyramidal geometry. The DFT and NMR results also reveal that antiferromagnetic coupling occurs between the two metal centers of the 2:1 complex observed in aqueous solution. These results are anticipated to provide crucial structural data relevant for subsequent studies of the physiological effects of the IC food dye. These studies will certainly be important in determining the origin of the reported side effects related to exposure to IC. 

## Figures and Tables

**Table 1:** Symmetries, relative Gibbs energies (ΔG_298K_) in kJ mol^−1^ at 298.15 K and equilibrium populations (P_298K_) in percentage, estimated from the relative Gibbs energies, calculated for the most stable forms of indigo carmine (B3LYP/6-311++G(d,p), taking into account the bulk solvent effects of water).

Structure	Symmetry	ΔG_298k_	P(%)_298K_
*trans*	C*_i_*	0.0	100.0
*cis*	C_2_	37.0	0.0
*trans T1*	C_1_	50.8	0.0
*cis T1*	C_1_	73.4	0.0

**Table 2:** Selected structural parameters of the *trans* isomer of indigo carmine calculated at the B3LYP/6-311++G(d,p) level taking into account the bulk solvent effects of water.

Bond Lengths (Å)		Angles (Degrees)	
O26⋯H36	2.305	H35-N23-C8	123.2
H35-N23	1.011	N23-C8-C19	126.1
N23-C8	1.378	C7-C8-C19	125.8
C8-C19	1.361	C8-C7-26	125.0
C7-C8	1.494	C7-C8-C19-C16	180.0
C1-C7	1.464	N23-C8-C7-C1	0.0
C7-O26	1.231	C8-C7-O26-H36	0.0
C1-C2	1.412	C19-C8-N23-H35	0.0
C2-C3	1.396	C3-C4-C5-C6	0.0

**Table 3:** Experimental ^1^H NMR chemical shifts (*δ*/ppm) (in H_2_O/D_2_O) for the free ligand (IC) and complexes **a**, **b** and **c** (atom numbering as shown in Scheme 1).

*δ*^1^H RMN (exp.) ^a^				
	Free Ligand	Complex a	Complex b	Complex c
H-36	- ^b^	- ^b^	- ^b^	- ^b^
H-11/H-34	7.90	7.98	7.89	8.84
H-10/H-20	7.58	7.80	7.64	8.67
H-9/H-21	6.66	7.08	6.90	8.26

^a^ *δ* values, in ppm, relative to Me_4_Si, using *tert*-butyl alcohol (*δ*_H_ = 1.2) as internal reference. ^b^ not detected.

**Table 4:** EDS results for the solid-state Cu(II):IC complex.

Element ^a^	Weight %	Atomic %	Calc. wt. % 1:2 Structure	Calc. wt. % 2:3 Structure
Carbon	30.9	44.7	35.6	37.6
Nitrogen	7.5	9.4	5.6	5.5
Oxygen	28.1	30.5	27.3	27.1
Sodium	6.9	5.2	9.2	9.0
Sulfur	9.9	5.4	12.9	12.5
Copper	15.8	4.3	6.4	8.3

^a^ Trace amounts of Al (0.08%), Si (0.48%), Cl (0.26%) and K (<0.01%) were also found.

## Data Availability

The original contributions presented in the study are included in the article/Supplementary Material; further inquiries can be directed to the corresponding author.

## Supplementary Materials

The following supporting information can be downloaded at https://www.mdpi.com/article/10.3390/molecules29133223/s1, Figure S1: DFT B3LYP/6-311++G(d,p) optimized geometries of the higher energy tautomers of indigo carmine in water, Figure S2: DFT B3LYP/6-311++G(d,p) calculated potential energy profile for conversion between the trans and cis conformers (dihedral angle C7-C8-C19-C16) of the indigo carmine molecule, Figure S3: Determination of a scaling factor for the calculated vibrational frequencies of IC from the linear regression between selected experimental frequencies observed in the FTIR-ATR spectrum of the pure IC and the respective B3YP/6-311++G(d,p) calculated frequencies, Figure S4: ATR-FTIR spectra (4000–2700/1800–400 cm^−1^) of the solid samples obtained from aqueous solutions of CuCl_2_·2H_2_O at pH 10.11 (top), Cu(II):IC 0.010:0.010 mol dm^−3^ at pH 9.95 (middle) and IC at pH 9.95 (bottom), Figure S5: ATR-FTIR spectra (1800–400 cm^−1^) of the solid samples obtained from aqueous solutions of CuCl_2_·2H_2_O:IC 10:10, 5:10 and 10:5 mmol dm^−3^, at pH 8.11, 7.95 and 8.00, respectively, Figure S6: (a) Absorption spectra of an IC:Cu(II) 1 × 10^−5^:1 × 10^−4^ mol dm^−3^ aqueous solution at pH 2.08, 4.13, 6.13, 6.97, 7.84, 8.99 and 9.97; (b) absorbance intensity at 610 nm (black squares) and 707 nm (red circles) as a function of pH, Figure S7: ^1^H NMR spectra of D_2_O solutions of (a) IC 5.0 mmol dm^−3^, pH* 8.05; (b) Cu(NO_3_)_2_:IC 10:5.0 mmol dm^−3^, pH* 7.60; (c) CuCl_2_:IC 20:5.0 mmol dm^−3^, pH* 8.05; (d) Cu(NO_3_)_2_:IC 20:5.0 mmol dm^−3^, pH* 8,11; Temp. 298.15 K, Figure S8: (a) ^1^H and (b) ^13^C NMR spectra of a 5 mmol dm^−3^ solution of IC in D_2_O, pH* 8.05, temp. 298.15 K, Figure S9: HSQC 2D-NMR spectra of a 5 mmol dm^−3^ solution of IC in D_2_O, pH* 8.05, temp. 298.15 K, Figure S10: HMBC 2D-NMR spectra of a 5 mmol dm^−3^ solution of IC in D_2_O, pH* 8.05, temp. 298.15 K, Figure S11: Main contribution to the excited state S1 of indigo carmine, which corresponds to the experimental band observed at 610 nm (TD-DFT/CAM-B3LYP, in water), Figure S12: Main contributions to the excited state predicted at 592 nm for the 1:2 Cu(II):IC complex (TD-DFT/CAM-B3LYP, in water, Figure S13: EPR spectra of (a) 2.5:5 mmol dm^−3^ and (b) 10:10 mmol dm^−3^ Cu(II):IC aqueous solutions at pH 8 (room temperature), Figure S14: IR calculated spectra (1800–400 cm^−1^) for the 1:1, 1:2, 2:1 singlet and 2:1 triplet structures (top to bottom, respectively) in comparison with the ATR-FTIR experimental spectrum of the solid powder obtained from a Cu(II):IC 5:10 mmol dm^−3^ aqueous solution at pH 8 (bottom). The vibrational frequencies of the theoretical spectra were scaled with the factors 0.976 for the 1:1 structure and 0.986 for the 1:2 and 2:1 structures, Figure S15: Determination of a scaling factor for the calculated vibrational frequencies of the 1:2 Cu(II):IC complex, from the linear regression between the experimental frequencies observed in the FTIR-ATR spectrum of the solid powder sample obtained from a Cu(II):IC 5:10 mmol dm^−3^ aqueous solution and the respective B3YP/6-311++G(d,p) calculated frequencies, Figure S16: ATR-FTIR spectrum (1800–400 cm^−1^) of the solid powder sample obtained from a Cu(II):IC 5:10 mmol dm^−3^ aqueous solution at pH 8 (top), in comparison with the DFT/B3LYP calculated spectrum for the 2:3 Cu(II):IC structure (bottom). The vibrational frequencies of the theoretical spectrum were scaled with the factor 0.986, Figure S17: Optimized geometry of the 2:3 Cu(II)/IC structure (optimized at the B3LYP/LanL2DZ/6-311++G(d,p) level of theory in water), Table S1: Relative Gibbs energies (kJ mol^−1^) at 298.15 K (ΔG_298K_) and equilibrium populations (%) estimated from the relative Gibbs energies (P_298K_), calculated for the higher energy tautomers of indigo carmine (B3LYP/6-311++G(d,p) in water), Table S2: Vertical excitation energies, oscillator strengths (*f*), wavelengths (λ) and major contributions calculated for the excited states of IC and Cu(II):IC 1:2 e 2:1 singlet complexes (TD-DFT CAM-B3LYP/6-311++G(d,p)).
