# Supplementary material for: Indigo Carmine Binding to Cu(II) in Aqueous Solution and Solid State: Full Structural Characterization Using NMR, FTIR and UV/Vis Spectroscopies and DFT Calculations
Source: Molecules. 2024 Jul 7;29(13):3223. doi: 10.3390/molecules29133223 (PMC11243005; doi:10.3390/molecules29133223)
Supplement: Supplementary file 1 [file molecules-29-03223-s001.zip › molecules-3001566-supplementary.pdf]

Supplementary Material

Indigo Carmine Binding to Cu(II) in Aqueous Solution and Solid State: Full Structural Characterization Using NMR, FTIR and UV/Vis Spectroscopies and DFT Calculations

Sofia Braz,[1,2] Licínia L. G. Justino,[1,*] M. Luísa Ramos[1] and Rui Fausto[1,3]

1 CQC-IMS, Department of Chemistry, University of Coimbra, Rua Larga, 3004-535 Coimbra, Portugal

2 CERES, Department of Chemical Engineering, University of Coimbra, Pólo II, Rua Sílvio Lima, 3030-790 Coimbra, Portugal

3 Istanbul Kultur University, Faculty of Sciences and Letters, Department of Physics, 34158 Bakirkoy, Istanbul, Turkey

* Correspondence: liciniaj@ci.uc.pt

Index:

Fig. S1. DFT B3LYP/6-311++G(d,p) optimized geometries of the higher energy tautomers of indigo carmine in water........ 3

Fig. S2. DFT B3LYP/6-311++G(d,p) calculated potential energy profile for conversion between the *trans* and *cis* conformers (dihedral angle C7-C8-C19-C16) of the indigo carmine molecule........ 4

Fig. S3. Determination of a scaling factor for the calculated vibrational frequencies of IC from the linear regression between selected experimental frequencies observed in the FTIR-ATR spectrum of the pure IC and the respective B3YP/6-311++G(d,p) calculated frequencies........ 4

Fig. S4. ATR-FTIR spectra (4000-2700/1800-400 cm^{-1}) of the solid samples obtained from aqueous solutions of $CuCl_2.2H_2O$ at pH 10.11 (*top*), Cu(II):IC 0.010:0.010 mol dm^{-3} at pH 9.95 (*middle*) and IC at pH 9.95 (*bottom*)........ 5

Fig. S5. ATR-FTIR spectra (1800-400 cm^{-1}) of the solid samples obtained from aqueous solutions of $CuCl_2.2H_2O$:IC 10:10, 5:10 and 10:5 mmol dm^{-3}, at pH 8.11, 7.95 and 8.00, respectively........ 5

Fig. S6. (a) Absorption spectra of a IC:Cu(II) $1x10^{-5}$:$1x10^{-4}$ mol dm^{-3} aqueous solution at pH 2.08, 4.13, 6.13, 6.97, 7.84, 8.99 and 9.97; (b) Absorbance intensity at 610 nm (*black squares*) and 707 nm (*red circles*) as a function of pH.................................. 6

Fig. S7. ^{1}H NMR spectra of D_2O solutions of: (a) IC 5.0 mmol dm^{-3}, pH* 8.05; (b) $Cu(NO3)_2$:IC 10:5.0 mmol dm^{-3}, pH* 7.60; (c) $CuCl_2$:IC 20:5.0 mmol dm^{-3}, pH* 8.05; (d) $Cu(NO_3)_2$:IC 20:5.0 mmol dm^{-3}, pH* 8,11; Temp. 298.15 K............................. 6

Fig. S8. (a) ^{1}H and (b) ^{13}C NMR spectra of a 5 mmol dm^{-3} solution of IC in D_2O, pH* 8.05, temp. 298.15 K... 7

Fig. S9. HSQC 2D-NMR spectra of a 5 mmol dm^{-3} solution of IC in D_2O, pH* 8.05, temp. 298.15 K.. 7

Fig. S10. HMBC 2D-NMR spectra of a 5 mmol dm^{-3} solution of IC in D_2O, pH* 8.05, temp. 298.15 K.. 8

Fig. S11. Main contribution to the excited state S_1 of indigo carmine, which corresponds to the experimental band observed at 610 nm (TD-DFT/CAM-B3LYP, in water)............. 8

Fig. S12. Main contributions to the excited state predicted at 592 nm for the 1:2 Cu(II):IC complex (TD-DFT/CAM-B3LYP, in water.. 9

Fig. S13. EPR spectra of a) 2.5:5 mmol dm-3 and b) 10:10 mmol dm-3 Cu(II):IC aqueous solutions at pH 8 (room temperature)...9

Fig. S14. IR calculated spectra (1800-400 cm^{-1}) for the 1:1, 1:2, 2:1 singlet and 2:1 triplet structures (*top* to *bottom*, respectively) in comparison with the ATR-FTIR experimental spectrum of the solid powder obtained from a Cu(II):IC 5:10 mmol dm^{-3} aqueous solution at pH 8 (*bottom*). The vibrational frequencies of the theoretical spectra were scaled with the factors 0.976 for the 1:1 structure and 0.986 for the 1:2 and 2:1 structures.. 10

Fig. S15. Determination of a scaling factor for the calculated vibrational frequencies of the 1:2 Cu(II):IC complex, from the linear regression between the experimental frequencies observed in the FTIR-ATR spectrum of the solid powder sample obtained from a Cu(II):IC 5:10 mmol dm-3 aqueous solution and the respective B3YP/6-311++G(d,p) calculated frequencies... 10

Fig. S16. ATR-FTIR spectrum (1800-400 cm^{-1}) of the solid powder sample obtained from a Cu(II):IC 5:10 mmol dm^{-3} aqueous solution at pH 8 (*top*), in comparison with the DFT/B3LYP calculated spectrum for the 2:3 Cu(II):IC structure (*bottom*). The vibrational frequencies of the theoretical spectrum were scaled with the factor 0.986...11

Fig. S17. Optimized geometry of the 2:3 Cu(II)/IC structure (optimized at the B3LYP/LanL2DZ/6-311++G(d,p) level of theory in water)………………………… 1

Table S1. Relative Gibbs energies (kJ mol^{-1}) at 298.15 K (ΔG_{298K}) and equilibrium populations (%) estimated from the relative Gibbs energies (P_{298K}), calculated for the higher energy tautomers of indigo carmine (B3LYP/6-311++G(d,p) in water)……………………... 12

Table S2. Vertical excitation energies, oscillator strengths (*f*), wavelengths (λ), and major contributions calculated for the excited states of IC and Cu(II):IC 1:2 e 2:1 singlet complexes (TD-DFT CAM-B3LYP/6-311++G(d,p))………………………………... 12

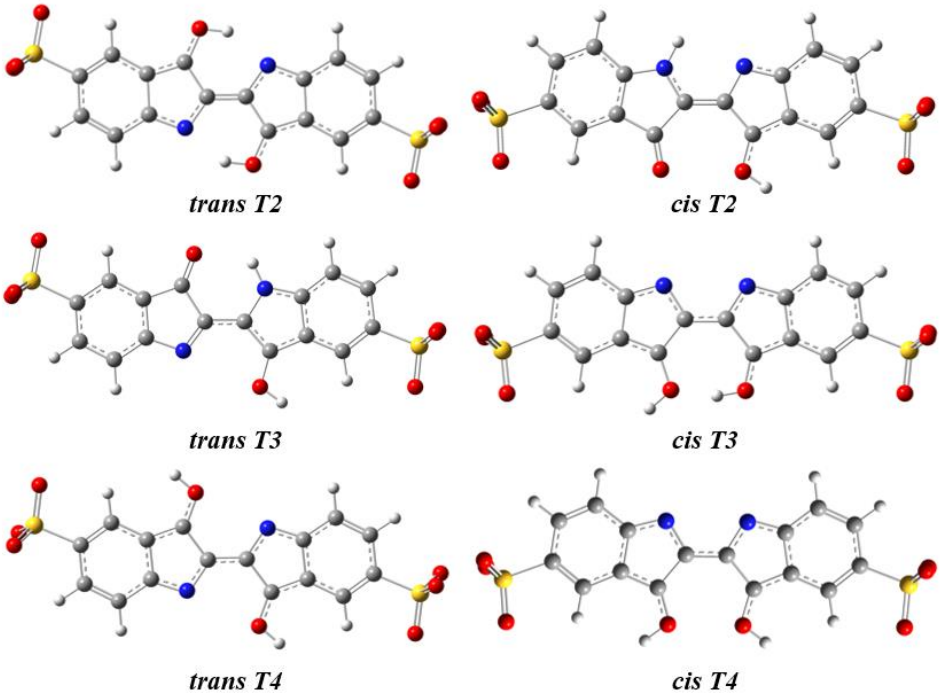

Figure S1. DFT B3LYP/6-311++G(d,p) optimized geometries of the higher energy tautomers of indigo carmine in water.

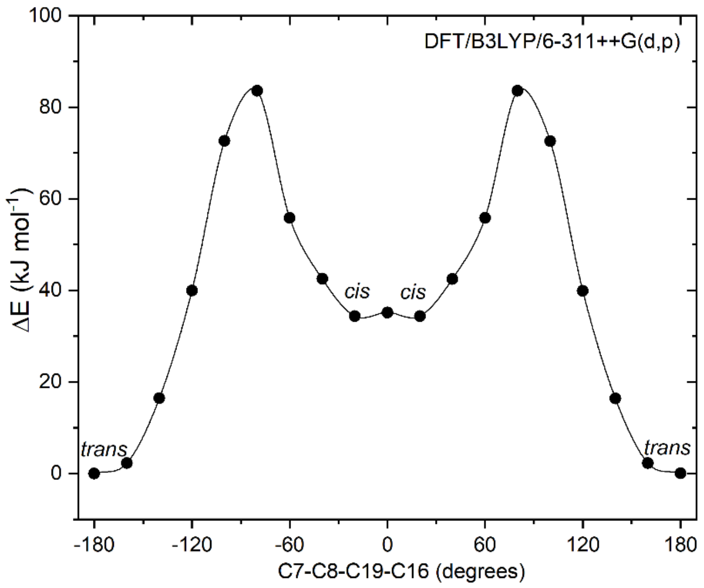

Figure S2. DFT B3LYP/6-311++G(d,p) calculated potential energy profile for interconversion between the *trans* and *cis* conformers (dihedral angle C7-C8=C19-C16) of the indigo carmine molecule (most stable tautomer).

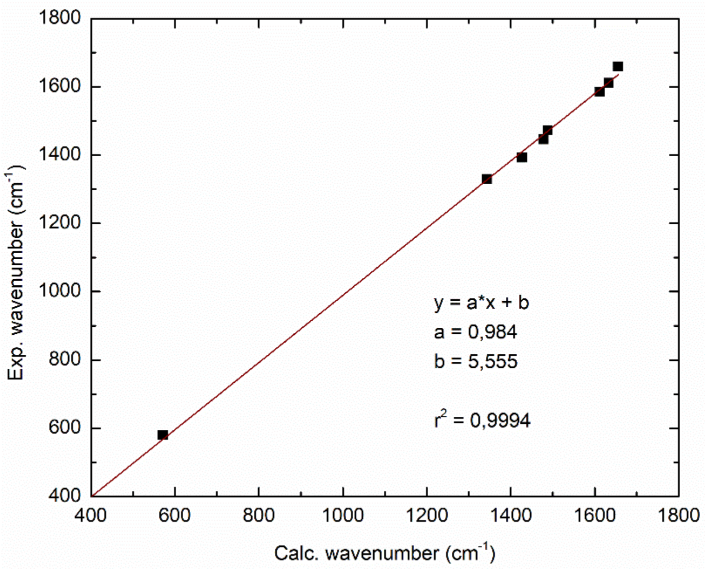

Figure S3. Determination of a scaling factor for the calculated vibrational frequencies of IC from the linear regression between selected experimental frequencies observed in the FTIR-ATR spectrum of the pure IC and the respective B3YP/6-311++G(d,p) calculated frequencies.

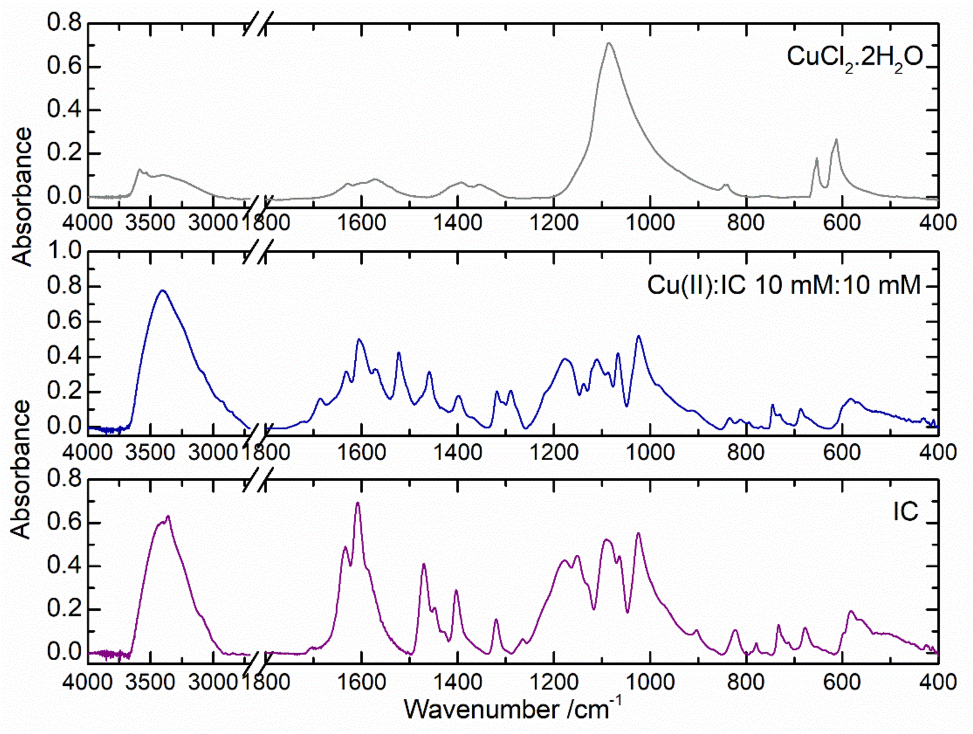

Figure S4. ATR-FTIR spectra (4000-2700/1800-400 cm^{-1}) of the solid samples obtained from aqueous solutions of $CuCl_2.2H_2O$ at pH 10.11 (*top*), Cu(II):IC 0.010:0.010 mol dm^{-3} at pH 9.95 (*middle*) and IC at pH 9.95 (*bottom*).

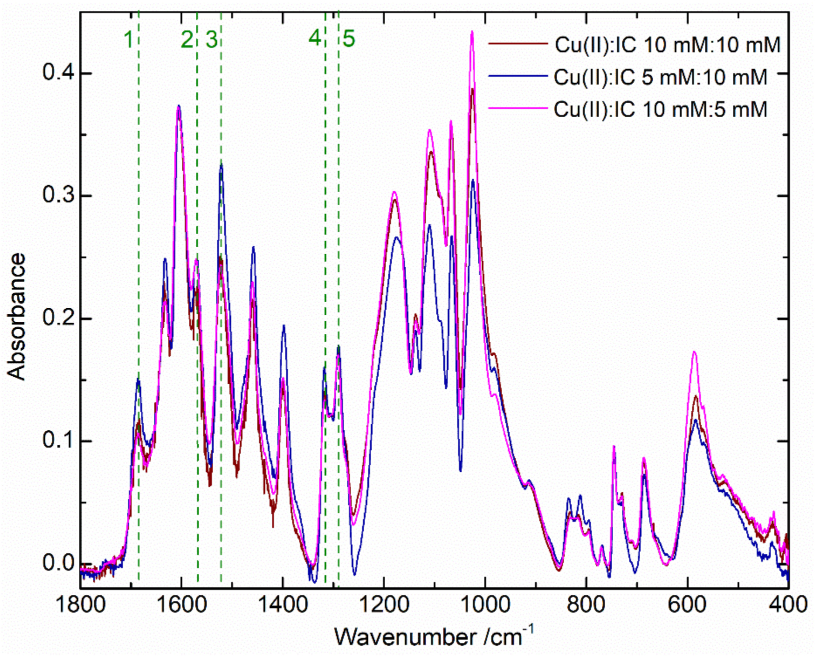

Figure S5. ATR-FTIR spectra (1800-400 cm^{-1}) of the solid samples obtained from aqueous solutions of $CuCl_2.2H_2O$:IC 10:10, 5:10 and 10:5 mmol dm^{-3}, at pH 8.11, 7.95 and 8.00, respectively.

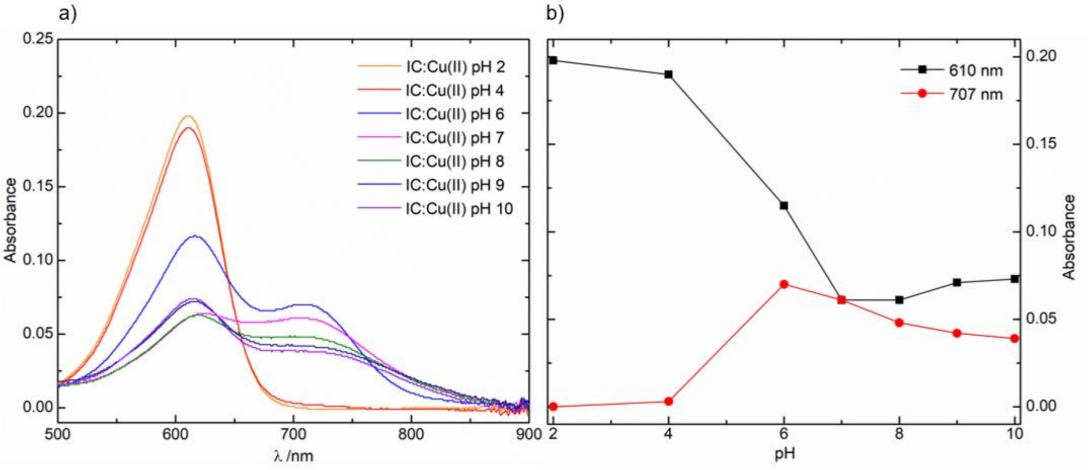

Figure S6. (a) Absorption spectra of a IC:Cu(II) $1x10^{-5}$:$1x10^{-4}$ mol dm^{-3} aqueous solution at pH 2.08, 4.13, 6.13, 6.97, 7.84, 8.99 and 9.97; (b) Absorbance intensity at 610 nm (*black squares*) and 707 nm (*red circles*) as a function of pH.

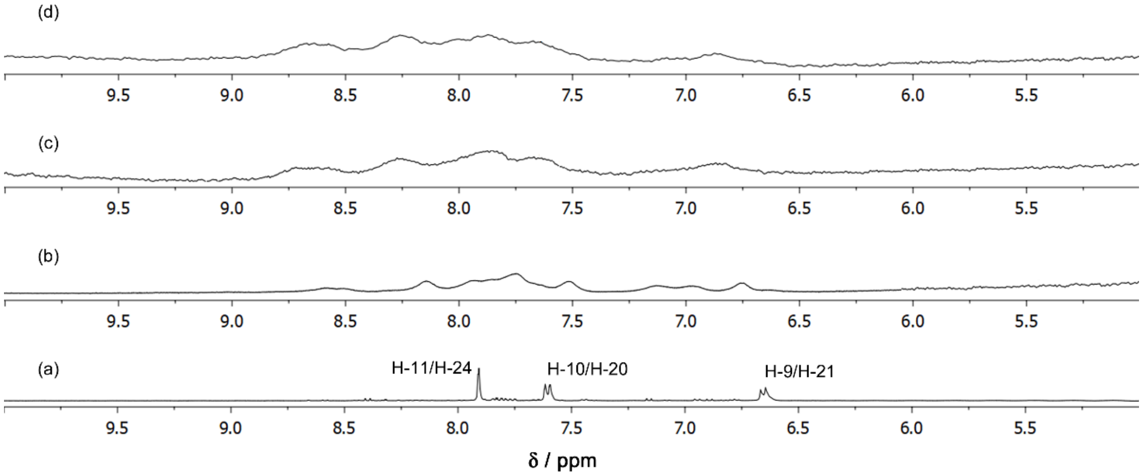

Figure S7. ^{1}H NMR spectra of D_2O solutions of: (a) IC 5.0 mmol dm^{-3}, pH* 8.05; (b) $Cu(NO3)_2$:IC 10:5.0 mmol dm^{-3}, pH* 7.60; (c) $CuCl_2$:IC 20:5.0 mmol dm^{-3}, pH* 8.05; (d) $Cu(NO_3)_2$:IC 20:5.0 mmol dm^{-3}, pH* 8,11; Temp. 298.15 K.

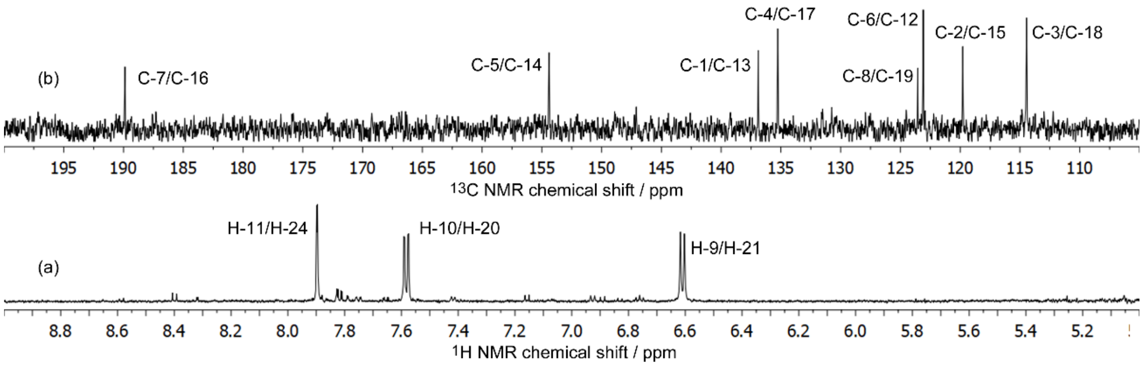

Figure S8. (a) ^{1}H and (b) ^{13}C NMR spectra of a 5 mmol dm^{-3} solution of IC in D_2O, pH* 8.05, temp. 298.15 K.

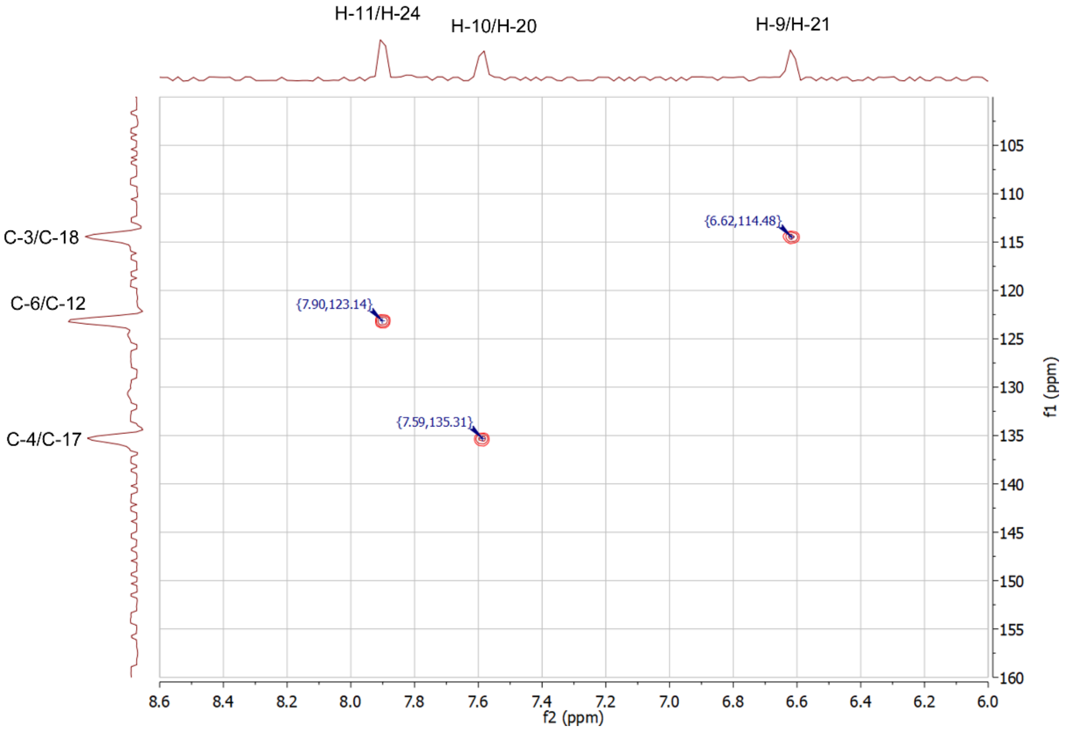

Figure S9. HSQC 2D-NMR spectra of a 5 mmol dm^{-3} solution of IC in D_2O, pH* 8.05, temp. 298.15 K.

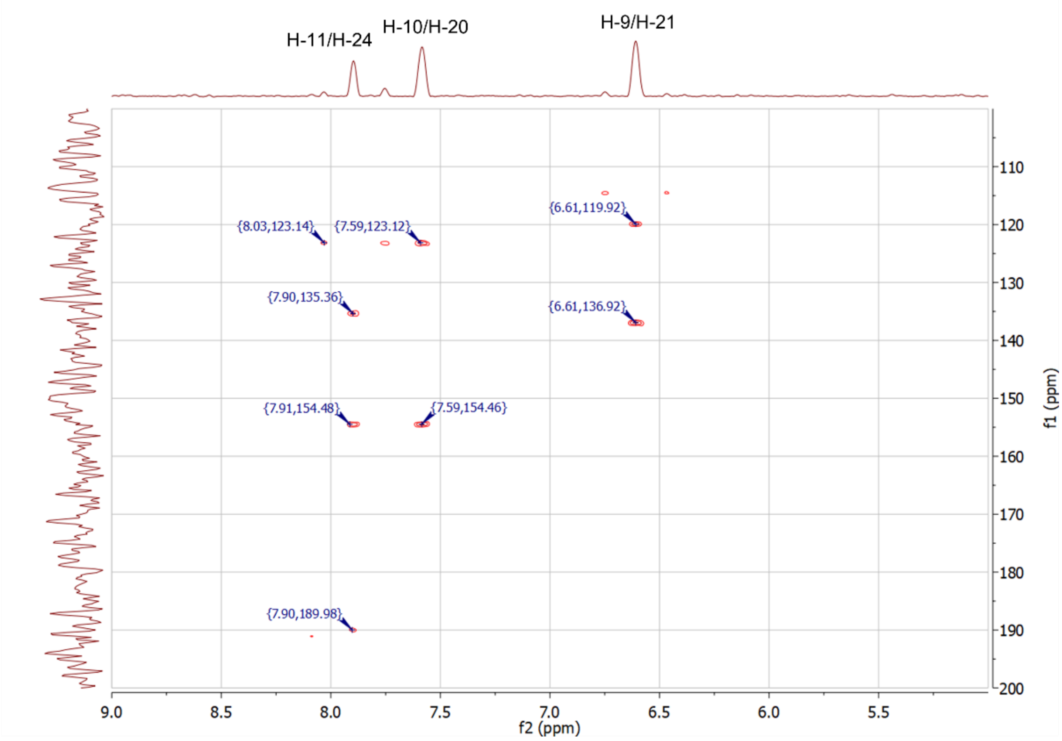

Figure S10. HMBC 2D-NMR spectra of a 5 mmol dm^{-3} solution of IC in D_2O, pH* 8.05, temp. 298.15 K.

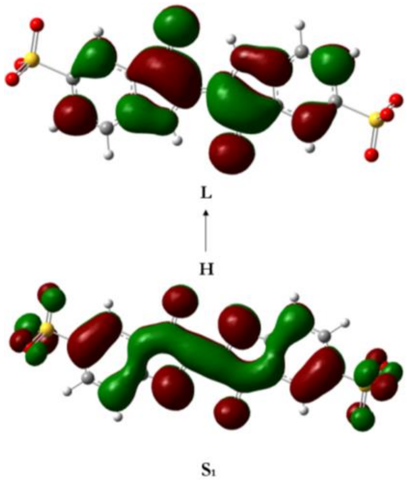

Figure S11. Main contribution to the excited state S_1 of indigo carmine, which corresponds to the experimental band observed at 610 nm (TD-DFT/CAM-B3LYP, in water).

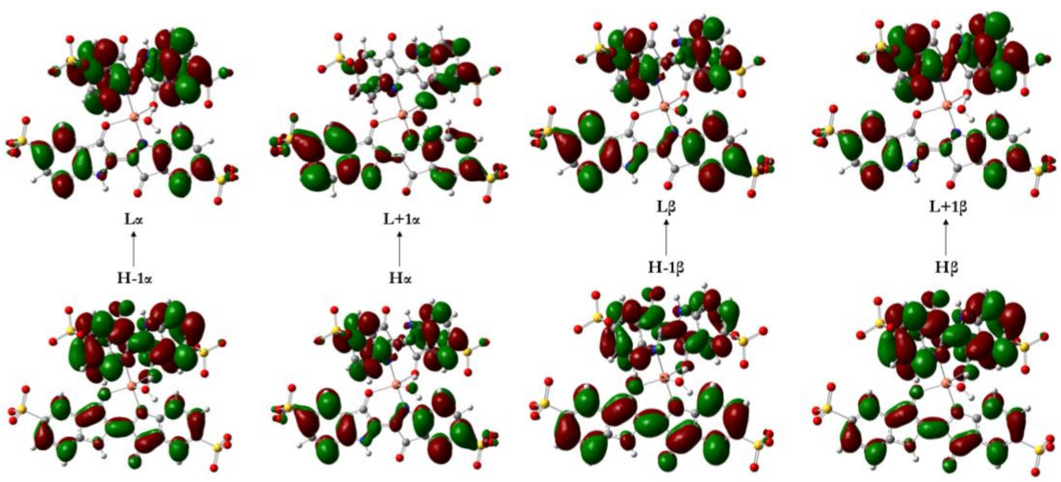

Figure S12. Main contributions to the excited state predicted at 592 nm for the 1:2 Cu(II):IC complex (TD-DFT/CAM-B3LYP, in water).

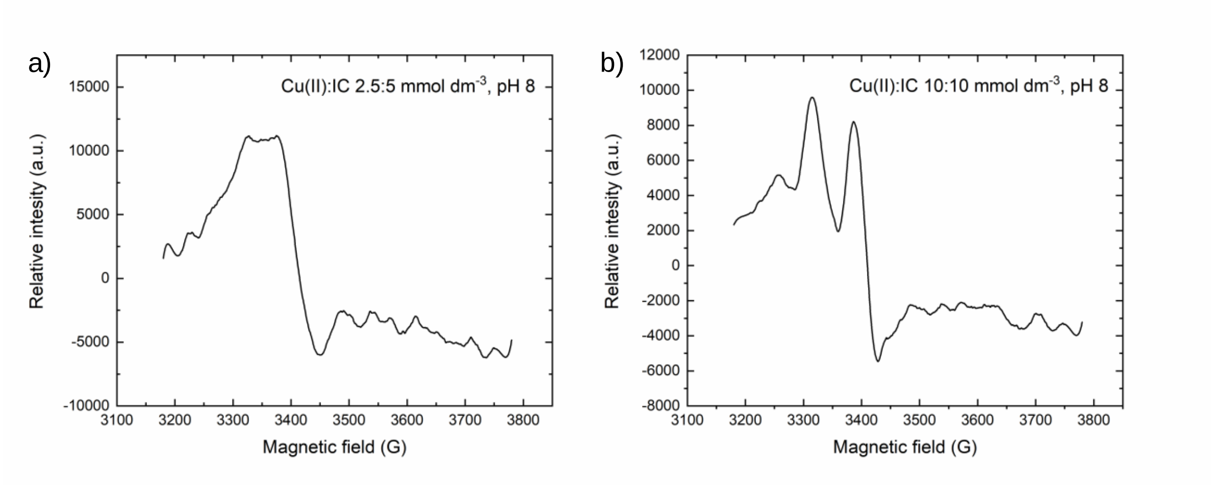

Figure S13. EPR spectra of a) 2.5:5 mmol dm^{-3} and b) 10:10 mmol dm^{-3} Cu(II):IC aqueous solutions at pH 8 (room temperature).

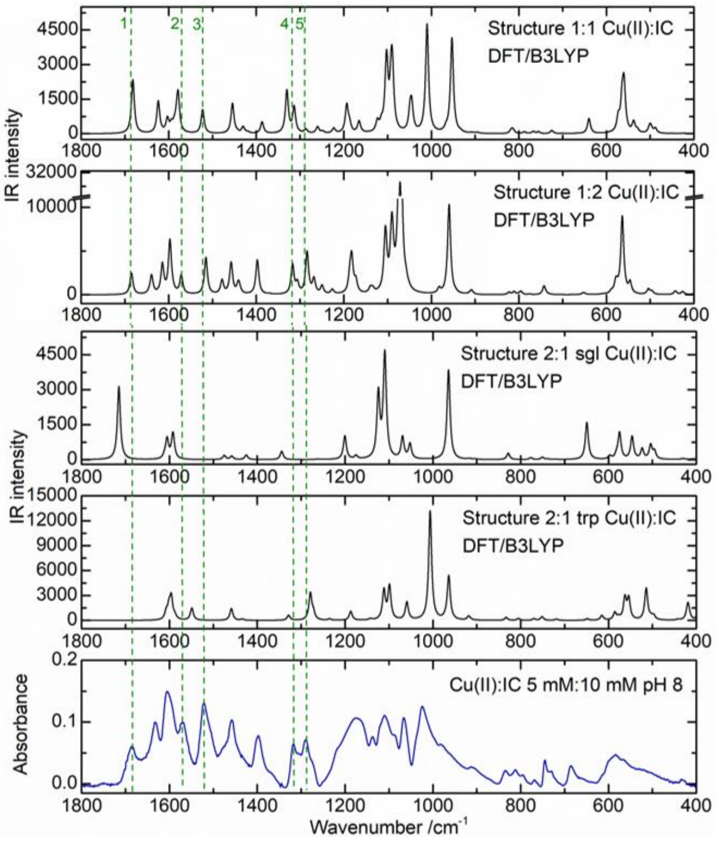

Figure S14. IR calculated spectra (1800-400 cm^{-1}) for the 1:1, 1:2, 2:1 singlet and 2:1 triplet structures (*top* to *bottom*, respectively) in comparison with the ATR-FTIR experimental spectrum of the solid powder obtained from a Cu(II):IC 5:10 mmol dm^{-3} aqueous solution at pH 8 (*bottom*). The vibrational frequencies of the theoretical spectra were scaled with the factors 0.976 for the 1:1 structure and 0.986 for the 1:2 and 2:1 structures.

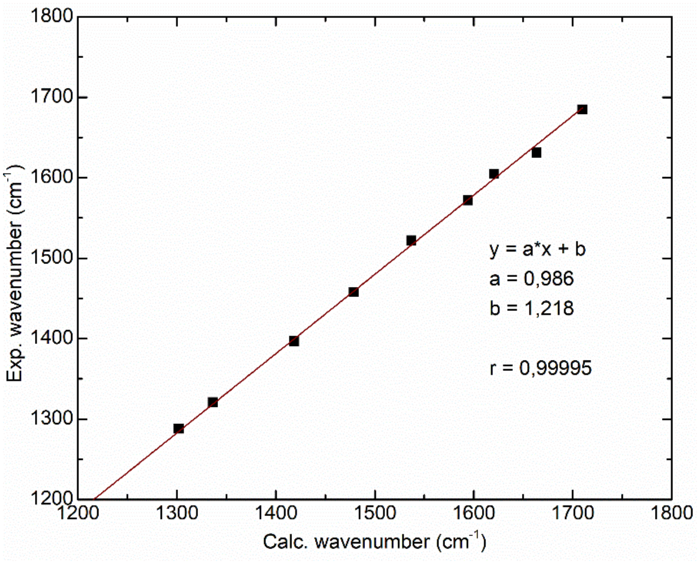

Figure S15. Determination of a scaling factor for the calculated vibrational frequencies of the 1:2 Cu(II):IC complex from the linear regression between the experimental frequencies observed in the FTIR-ATR spectrum of the solid powder sample obtained from a Cu(II):IC 5:10 mmol dm^{-3} aqueous solution and the respective B3YP/6-311++G(d,p) calculated frequencies.

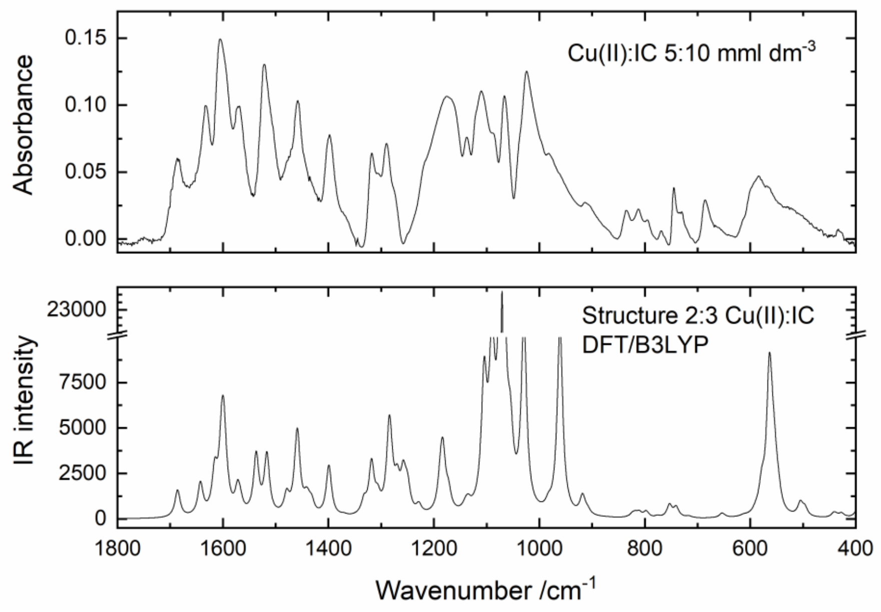

Figure S16. ATR-FTIR spectrum (1800-400 cm^{-1}) of the solid powder sample obtained from a Cu(II):IC 5:10 mmol dm^{-3} aqueous solution at pH 8 (*top*), in comparison with the DFT/B3LYP calculated spectrum for the 2:3 Cu(II):IC structure (*bottom*). The vibrational frequencies of the theoretical spectrum were scaled with the factor 0.986.

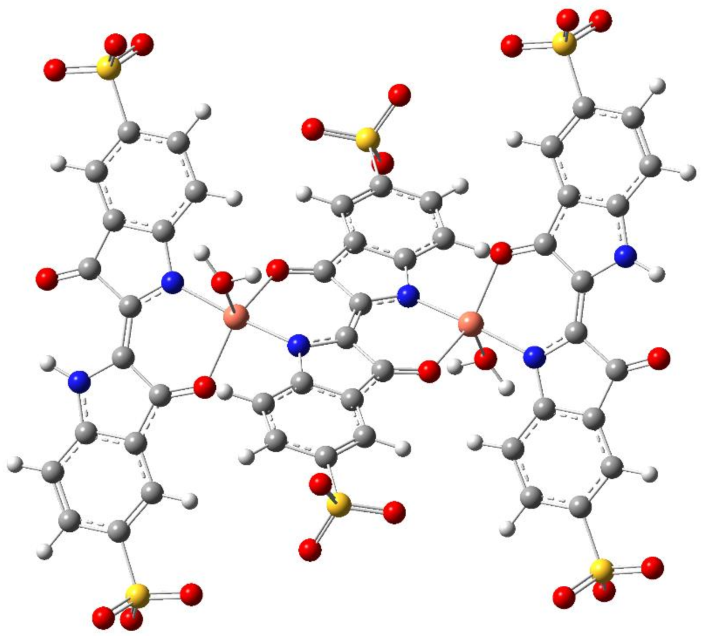

Figure S17. Optimized geometry of the 2:3 Cu(II)/IC structure (optimized at the B3LYP/LanL2DZ/6-311++G(d,p) level of theory in water).

Table S1. Relative Gibbs energies (kJ mol^{-1}) at 298.15 K (ΔG_{298K}) and equilibrium populations (%) estimated from the relative Gibbs energies (P_{298K}), calculated for the higher energy tautomers of indigo carmine (B3LYP/6-311++G(d,p) in water).

**Structure**	**Symmetry**	**ΔG_{298k}**	**$P(\%)_{298K}$**
trans T2	C_i	739.7	0.0
trans T3	C_1	74.9	0.0
trans T4	C_i	234.6	0.0
cis T2	C_1	116.7	0.0
cis T3	C_1	216.0	0.0
cis T4	C_2	241.4	0.0

Table S2. Vertical excitation energies, oscillator strengths (*f*), wavelengths (λ), and major contributions calculated for the excited states of IC and Cu(II):IC 1:2 e 2:1 singlet complexes (TD-DFT CAM-B3LYP/6-311++G(d,p)).

Energy (eV)	$\lambda_{calc.}$[a] (nm)	$\lambda_{exp.}$ (nm)	*F*	Major contributions (%)[b]
			**Indigo Carmine**	
2.37	522	610	0.458	H → L (100%)
			**1:2 complex**	
1.69	735	-[c]	0.006	H-39β → L+2β (48%)
2.02	615		0.083	H-1α → L+1α (15%) +
				Hα → Lα (24%) +
				H-1β → L+1β (14%) +
		710		Hβ → Lβ (25%)
2.10	592		0.590	H-1α → Lα (16%) +
				Hα → L+1α (24%) +
				H-1β → Lβ (15%) +
				Hβ → L+1β (25%)
			**2:1 singlet complex**	
1.97	629	-[c]	0.228	H → L (97%)
2.28	545	-[c]	0.001	H-8 → L (100%)
2.40	517		0.008	H-11 → L (21%) +
				H-10 →L (67%) +
		-[c]		H-4 → L (12%)
2.45	505		0.012	H-11 → L (77%) +
				H-10 → L (10%)

[a] The results refer to the 500 – 900 nm region.
[b] Only contributions ≥ 10% are included.
[c] Not observed.
